# A Dithio Vinylthio C_2_ Synthon Enabling Crystalline and Luminescent Sulfur‐Decorated Polymers

**DOI:** 10.1002/anie.202525801

**Published:** 2026-02-15

**Authors:** Bercis Pektas, Cuong M. Q. Le, Samar Hajjar‐Garreau, Simon Gree, Hatice Mutlu

**Affiliations:** ^1^ Department of Chemistry, Technical Polymer Chemistry Rheinland‐Pfälzische Technische Universität Kaiserslautern‐Landau (RPTU) Kaiserslautern Germany; ^2^ Institut de Science des Matériaux de Mulhouse UMR 7361 CNRS/Université de Haute Alsace Mulhouse Cedex France; ^3^ Leibniz‐Institut für Verbundwerkstoffe (IVW) GmbH Erwin‐Schrödinger‐Straße 58 Kaiserslautern Germany

**Keywords:** α,ω‐Bis(Vinylthio) Monomers, C_2_‐Type Molecular Unit, Modular Polymer Design, Sulfur‐Decorated Polymers

## Abstract

While thioether linkages are commonly associated with soft and amorphous polymer backbones, herein, we show that a calcium carbide‐derived α,ω‐bis(vinylthio) synthon enables crystalline, sequence‐defined sulfur polymers with nonconventional luminescence. A single‐step thiol–yne addition reaction uses acetylene generated in situ from industrial calcium carbide (CaC_2_) to produce a modular C_2_ vinyl sulfide (also known as vinylthio) monomer that undergoes quantitative, light‐induced thiol‐ene step‐growth polymerization with aliphatic dithiols. Type I photoinitiation ensures complete anti‐Markovnikov addition to give C_2_ ‐segmented poly(thioether)s, while thermal and base‐mediated conditions generate hybrid poly(thioether)/polydisulfide structures. The resulting sulfur‐rich backbones display sharp melting transitions, spherulitic crystallization, one‐step thermal decomposition up to about 316°C, and pronounced cluster‐triggered emission arising from dense thioether clustering and through‐space conjugation. Green metric analysis reveals high Atom Economy and essentially waste‐free polymer formation, thereby linking efficient use of an established C_2_ synthon to precision sulfur polymer design. This C_2_ vinylthio platform provides a general strategy to convert classically soft thioether motifs into structurally ordered and luminescent materials, and establishes vinyl sulfides as powerful, yet underutilized, building blocks in sustainable polymer chemistry.

## Graphical abstract

Transforming CaC_2_ into a modular C_2_ building block enables the synthesis of α,ω‐bis(vinylthio) monomer and its conversion into crystalline, sequence‐defined sulfur‐decorated polymers. Their dense thioether clustering induces nonconventional luminescence and provides structure–function control in a more‐sustainable polymer platform.

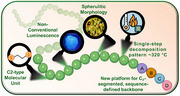

Over the past few decades, sulfur‐decorated polymers have attracted considerable attention in both academic and industrial research due to their exceptional optical, adhesive, and recyclable characteristic, as well as their strong coordination to metal ions [1, 2]. Their structural diversity spans thioethers, disulfides, thioesters, thiocarbonates, thioamides, and related motifs [1], with thioether linkages in particular imparting flexibility and tunability relative to oxygen analogues [2, 3]. Thioethers are generally associated with soft, conformationally mobile chains that yield amorphous materials. Nevertheless, their high polarizability and van der Waals interactions can, under appropriate spacing, promote intermolecular order and crystallinity [4, 5, 6, 7, 8, 9]. In addition, the electron‐rich nature of sulfur can induce cluster‐triggered emission (CTE) via through‐space conjugation (TSC), endowing sulfur‐decorated polymers with unusual photophysical behavior led by the electron‐rich character of sulfur [10, 11, 12, 13, 14, 15, 16, 17]. Taken together, these structural and photophysical attributes make sulfur‐decorated polymers promising candidates for advanced coatings, adhesives, and luminescent materials [8, 9, 10, 11, 12, 13, 14, 15, 16, 18]. While thiol‐ene chemistry stands as one of the most versatile and sustainable tools for constructing sulfur‐decorated polymers [19], its efficiency is strongly influenced by the nature of the alkene. Electron‐rich alkenes such as vinyl ethers exhibit high reactivity toward thiyl radicals, whereas electron‐deficient alkenes, including acrylates and maleimides, react much more slowly. In contrast, their sulfur analogues, vinyl sulfides [20, 21, 22, 23, 24, 25] (also known as vinyl thioethers, i.e., alkenes bearing a vinyl group directly attached to the sulfur, CH_2_ = CH‐S‐R), have received comparatively little attention, mainly due to misconceptions regarding their stability and synthetic accessibility. Unlike vinyl ethers, the β‐carbon of a vinyl sulfide lacks sufficient electrophilicity to support polar thiol–Michael additions, ensuring that thiol–ene polymerization proceeds exclusively through a radical, anti‐Markovnikov pathway under all practical conditions. Against this background, while monofunctional α‐(vinylthio) monomers have been synthesized (see Scheme 1) and polymerized successfully by free radical and RAFT methods, as showcased by Brendel and co‐workers [26], their bifunctional counterparts, α,ω‐bis(vinyl sulfide) (α,ω‐bis(vinylthio)) monomers (shown in Scheme 2b) have remained largely unexplored and scientifically dormant [25, 27], awaiting systematic revival and understanding. Placing two vinyl sulfide termini on a single scaffold provides a straightforward design logic for step‐growth thiol–ene polymerization with dithiols. We reasoned that such a polyaddition would install a regular ethylene (C_2_) spacer between adjacent thioether units, yielding sulfur‐decorated backbones with intrinsic sequence definition (see Scheme 2c). Indeed, comparable C_2_ spacers have previously been realized through dithiol–dibromide polycondensations, for instance, the reaction between diverse dimercaptans and 1,2‐dibromoethane [18], although such approaches typically require harsh conditions and often suffer from poor selectivity and side reactions. Related sulfur‐rich polymer backbones with regular C_2_ spacers have also been accessed via the ring‐opening polymerization of thiiranes, as reported in early and more recent studies on functional sulfur‐containing materials [28, 29, 30, 31]. In contrast, α,ω‐bis(vinyl sulfide) monomers offer a milder, catalyst‐controlled, and atom‐efficient pathway to achieve comparable structural precision. This periodic C_2_ spacing is anticipated to influence chain packing, polarity, and intermolecular interactions, providing a direct handle on chain order and crystallinity. In this sense, α,ω‐bis(vinyl sulfide) monomers establish a conceptual bridge between molecular reactivity and material structure, serving as a minimal, yet powerful motif for constructing sequence‐defined sulfur polymers [26]. To access such building blocks in a concise manner, we developed a scalable method that uses acetylene generated in situ from calcium carbide (CaC_2_) as a solid C_2_ synthon. CaC_2_ [32, 33, 34], produced on a large industrial scale, releases acetylene upon hydrolysis; the resulting acetylene undergoes a thiol–yne click reaction with dithiols under mild basic conditions, yielding the targeted α,ω‐bis(vinyl sulfide) monomers in a single step. This transformation takes advantage of a convenient acetylene carrier and achieves good Atom Economy under metal‐free and comparatively mild conditions. However, the overall environmental profile of the route remains governed by the energy‐intensive production of CaC_2_ and the management of carbide‐derived residues [33, 34, 35]. Accordingly, reaction of 1,6‐hexanedithiol (**DT_1_**) with acetylene gas, generated in situ from CaC_2_ hydrolysis, afforded the target α,ω‐bis(vinyl sulfide) monomer (**M1**) in 73% isolated yield (see Scheme 2 and Schemes S1, S2 in the Supporting Information). The selection of **DT_1_** was deliberate: its linear six‐carbon spacer represents the classical “C_6_” motif found in archetypal step‐growth polymers, such as polyamides [36] and polyesters, where the methylene sequence balances flexibility and regularity. Importantly, the overall synthetic strategy could readily accommodate shorter, longer, or heteroatom‐containing dithiols. The absence of branching or heteroatoms in the targeted monomer **M1** would minimize steric interference and ensure that the reactivity arises solely from the terminal thiol functions. This structural simplicity enables high‐yield monomer formation via thiol–yne addition and is expected to promote efficient chain alignment and crystallinity in the resulting polymers. In addition, **DT_1_** is commercially accessible, low in toxicity [37], and provides a defined intramolecular distance between thiols, serving as an ideal platform for exploring how C_2_‐periodic sulfur incorporation modulates chain order, packing, and optical behavior. The mild thiol–yne reaction proceeded cleanly, as verified by complementary spectroscopic analyses. Exemplary, in the ^1^H NMR spectrum of **M1** (see Figure 1a), characteristic magnetic resonances of α‐ and β‐protons in the vinyl group were detected at 6.36 and 5.13 ppm, respectively. Complementary to the ^1^H NMR, the attenuated total reflectance infrared (ATR‐IR) spectrum of **M1** depicted that the characteristic bands at 3091, 1086, 957.5, and 857.7 cm^−1^ arose from the stretching of = C‐H and bending of monosubstituted C = C adjacent to the thioether bond in **M1** (see Figure 1b). Raman spectroscopy, chosen for its sensitivity to sulfur vibrations, revealed distinct C–S–C stretching bands and no features corresponding to S–S or S = O species, confirming complete conversion and chemical purity. X‐ray photoelectron spectroscopy (XPS) corroborated this result, showing a single S 2p doublet at 163.5 eV characteristic of nonoxidized thioether sulfur. Crucially, these analyses were performed on the resulting polymers (vide infra), and the absence of S–S and S = O signatures, which would be expected to persist upon polymerization if formed during monomer synthesis, provides indirect support for a clean thiol–yne transformation. The combination of Raman and XPS thus provided orthogonal evidence for the chemical integrity of the sulfur‐containing motifs in the resulting materials. Vide supra, thiol–ene reactions are known to proceed through multiple mechanistic manifolds depending on how the thiyl radical (or thiolate) is generated. For vinyl sulfides, whose β‐carbon is less electrophilic than that of vinyl ethers, the corresponding reactivity profile has remained less established. Thus, the polymerization was first examined under neat, thermal conditions at 80°C [38], representing the classical initiation mode for thiol–ene systems (compare Section A3 in the Supporting Information), with **M1** and 1,6‐dithiol (i.e., **DT_1_**). A gradual decrease of vinyl resonances accompanied by the appearance of thioether signals confirmed the onset of radical addition (see Figure 2a). However, additional magnetic resonances at δ ≈ 2.8 ppm, attributable to methylene protons adjacent to disulfide linkages (–CH_2_–S–S–CH_2_–), together with modest molecular‐weight growth observed in the size‐exclusion chromatography (SEC) traces (see Figure 2b), indicated incomplete conversion even after prolonged heating for 24 h. Taken together, these features reveal that, under catalyst and solvent‐free thermal conditions, the desired thiol–ene propagation competes with thiyl radical recombination, yielding a mixed poly(thioether)/polydisulfide microstructure. To overcome the limitations of catalyst‐free, thermal initiation, a photochemical route was next explored. Irradiation of an equimolar mixture of **M1** and **DT_1_** in THF (1 M) at ambient temperature using 1.0 mol‐% diphenyl(2,4,6‐trimethylbenzoyl)phosphine oxide (TPO), a Type I α‐cleavage photoinitiator, at 365 nm afforded a rapid and clean reaction, reaching full vinyl conversion within 20 min (compare the NMR spectra of the monomer **M1** and **DT_1_** with the crude reaction mixture of the polymerization (**P1**) shown in Figure S2). Upon α‐cleavage, TPO [39, 40] generates benzoyl and phosphinyl radicals that efficiently abstract hydrogen from thiols, producing thiyl radicals that add selectively to the terminal carbon of the vinyl sulfide double bond. Comparison of the ^1^H NMR spectra of the monomer **M1** and the poly(thioether) (**P1**) reveals complete disappearance of olefinic resonances (observed at δ ≈ 6.2–6.7 ppm) and appearance of characteristic thioether signals at δ ≈ 2.7 ppm (indicated as a′ in Figure 1a), which are consistent with complete anti‐Markovnikov thiol–ene addition and, particularly, the formation of the (–S–CH_2_–CH_2_–S–) linkage featuring C_2_ spacing. The absence of any disulfide‐associated resonances has further highlighted the selectivity and clean radical propagation achieved under photochemical initiation. Upon precipitation of the reaction mixture into ice‐cold methanol, **P1** was isolated as a fine white powder in 90 % yield. Although polymerization proceeded homogeneously in THF, the isolated polymer exhibited markedly limited solubility in THF but remained readily soluble in chloroform. This behaviour is consistent with the development of enhanced interchain cohesion and crystalline order, in line with the anticipated C_2_‐segmented and sulfur‐rich structure of the polymer backbone. Thus, ^13^C NMR analysis of the precipitated polymer in CDCl_3_ further corroborates full vinyl bond consumption and uniform backbone structure (see Figure S3 in the Supporting Information). The vinyl carbon resonances at δ ≈ 128 ppm and 136 ppm vanish entirely, replaced by new aliphatic signals at δ ≈ 34 ppm corresponding to –S–CH_2_–CH_2_–S– linkages. Consistent with these findings, comparative ATR–IR spectra (see Figure 1b) of **M1** and **P1** revealed the loss of = C–H and C = C stretching bands and the appearance of strong C–S absorptions between 650–750 cm^−^^1^, further verifying complete thiol–ene conversion. XPS (vide supra) additionally confirmed the chemical composition and oxidation state of sulfur in poly(thioether) **P1**.  The survey spectrum revealed dominant peaks corresponding to carbon (C 1s at ∼284.8 eV) and sulfur (S 2p at ∼163.6 eV), with minor oxygen contributions arising from surface adsorption. The high‐resolution S 2p spectrum (see Figure S15 in the Supporting Information) displayed a well‐defined doublet with binding energies at 163.6 eV (S 2p_3_/_2_) and 164.8 eV (S 2p_1_/_2_), characteristic of thioether sulfur (C–S–C) environments. Importantly, no additional peaks at higher binding energies (typically ∼167–169 eV) were observed, ruling out the presence of oxidized sulfur species such as sulfoxides or sulfones. These results indicate that photochemical polymerization proceeds cleanly, preserving the divalent sulfur oxidation state throughout the reaction. The sharp S 2p doublet further supports a chemically uniform thioether environment, consistent with the ^1^H and ^13^C NMR spectra and the Raman results, which confirmed the absence of disulfide linkages (see Figure 3). Taken together, the XPS data indicate that **P1** (see Figure S15 in the Supporting Information) consists predominantly of nonoxidized thioether units, highlighting the high selectivity and mildness of light‐driven thiol–ene polymerization. Encouraged by these results, a base‐mediated polymerization pathway was next examined to evaluate whether a polar, thiolate‐driven mechanism could compete with radical addition. The reaction was carried out both under bulk conditions and in solution (i.e., THF) at ambient temperature using 0.1 mol% 1,8‐diazabicyclo[5.4.0]undec‐7‐ene (DBU) as a strong, non‐nucleophilic base (for details, compare section A3 in the Supporting Information). In analogy to previous reports [41] showing that base catalysis can promote thiol–ene addition with vinyl ethers through a thiol–Michael‐type process, this experiment sought to determine whether the sulfur analogue, vinyl sulfide, could exhibit comparable ionic reactivity, thereby serving as a mechanistic control. However, the β‐carbon of vinyl sulfide lacks sufficient electrophilicity to support nucleophilic attack by thiolate anions. Consistent with this expectation, ^1^H NMR analysis (see Figure 2c) revealed only partial consumption of vinyl resonances and the appearance of additional signals at δ ≈ 2.9 ppm, assignable to methylenes adjacent to disulfide linkages, indicating competing thiyl radical recombination. The SEC traces showed predominantly low‐molecular‐weight oligomers (see Figure 2d). These findings support that vinyl sulfides do not readily undergo ionic thiol–Michael reactions, and that polymerization proceeds via a radical anti‐Markovnikov thiol–ene pathway, as established under photochemical conditions. Encouraged by the efficiency and selectivity of the photochemical polymerization, the reaction scope was expanded to evaluate the generality of this α,ω‐bis(vinylthio)–thiol coupling approach. To this end, in addition to **DT_1_**, three structurally diverse dithiols, namely 2,2′‐thiodiethanethiol (**DT_2_**), 3,6‐dioxa‐1,8‐octanedithiol (**DT_3_**), and DL‐dithiothreitol (**DT_4_**), were investigated to elucidate the relationship between chain architecture and polymer properties. The selection of these dithiols was guided by rational structural variation: 1,6‐hexanedithiol offers a linear and flexible six‐carbon spacer that minimizes steric hindrance and ensures efficient radical propagation; 2,2′‐thiodiethanethiol introduces a thioether bridge that enhances sulfur density and potential intermolecular interactions; 3,6‐dioxa‐1,8‐octanedithiol incorporates ether linkages, allowing the introduction of polarity and conformational softness; and DL‐dithiothreitol, being renewable, introduces stereochemical complexity and pendant hydroxyl functionalities that can promote intermolecular hydrogen bonding. Together, these structural variations enable a systematic investigation of how backbone polarity, heteroatom content, and secondary functional groups influence polymer crystallinity and optical behaviour. All polymerizations proceeded smoothly under identical photochemical conditions, affording the corresponding poly(thioether)s (**P2–P4**) as white microcrystalline powders upon precipitation (see Figure 1a). ^1^H NMR spectra supported full anti‐Markovnikov addition through disappearance of the olefinic resonances and emergence of characteristic thioether methylene signals at δ ≈ 2.7 ppm, consistent with formation of the –S–CH_2_–CH_2_–S– motif (see Figure 1a). For **P3** and **P4**, additional 2D NMR, particularly, Heteronuclear Single Quantum Coherence (HSQC) spectra (see Figures S7 and S8 in the Supporting Information), underpinned the expected C–H correlations of the anti‐Markovnikov thioether linkages. Due to its very limited solubility, **P2** could not be analyzed by 2D NMR under the experimental conditions available, and this limitation is noted explicitly. Despite comparable conversion and selectivity, distinct differences have been observed in physical properties; for example, **P3** and **P4** have displayed partial solubility in chloroform and THF, while **P2** was insoluble in common organic solvents, consistent with stronger intermolecular cohesion through S···S interactions [42]. SEC measurements were performed for polymer samples that were sufficiently soluble under the SEC conditions available, that is, with THF as eluent. For these soluble samples, i.e., **P3** and **P4**, SEC (with polystyrene calibration) provided weight‐average molar masses (*M*_w_) in the range of approximately 8.000–9.000 g mol^−^^1^ with dispersity (*Ð*) values between 1.7 and 2.3 (see Figures S9 and S10 in the Supporting Information). The least soluble polymer, **P2,** did not yield a reliable SEC trace under any of the eluents or temperatures accessible in our laboratory. Accordingly, the SEC values reported here refer exclusively to soluble fraction of the samples. These findings highlight that the α,ω‐bis(vinylthio) platform enables the construction of sequence‐defined, C_2_‐segmented sulfur polymers whose morphology, crystallinity, and luminescence can be readily tuned by molecular architecture. ATR‐IR spectroscopy provided additional structural confirmation, with  **P2–P4** displaying characteristic absorption bands associated with the thioether backbone and the respective substituents (see Figures S12–S14 in the Supporting Information). The disappearance of C = C stretching vibrations and the appearance of ν(C–S) bands confirm the complete thiol–ene conversion and formation of thioether linkages. Notably, **P2**, which lacks oxygen‐containing motifs, shows a simplified fingerprint region dominated by thioether vibrations. **P4** exhibits additional signals near 1030–1270 cm^−^^1^ arising from C–O and C–S–C vibrations, consistent with the presence of heteroatoms (i.e., sulfur) and hydroxyl functionalities in its backbone. In contrast, **P3**, which incorporates also ether linkages, shows enhanced intensity in the 1100 cm^−^^1^ region due to ν(C–O–C) stretching, confirming successful incorporation of oxygen‐containing segments. Further structural confirmation was obtained from Raman spectroscopy analysis of **P1–P4** (see Figure 3), which have displayed distinct bands in the 580–780 cm^−^^1^ region, characteristic of C–S and CH–S stretching vibrations, consistent with the formation of thioether linkages. Specifically, all samples have exhibited intense features between 670 and 780 cm^−^^1^, assignable to C–S stretching, and weaker shoulders near 600 cm^−^^1^, corresponding to CH–S modes. Among the polymers, **P1** and **P2** displayed sharper and more intense C–S stretching bands, consistent with their higher degree of structural order and partial crystallinity, whereas **P3** and **P4** exhibited broader features indicative of increased conformational freedom and reduced chain packing. In addition, XPS S 2p spectra were recorded for **P2**, **P3**, and **P4** (see Figures S16–S18 in the Supporting Information). All three depicted a single main component in the binding energy range expected for reduced sulfur species; together with the Raman and NMR data, this supports thioether units as the main sulfur environment. These findings, in conjunction with the NMR and ATR‐IR data, have provided unambiguous evidence that the light‐induced polymerization has proceeded exclusively through the anti‐Markovnikov thioether‐forming pathway, affording poly(thioether)s largely devoid of oxidative or recombination‐derived defects.

**SCHEME 1:**
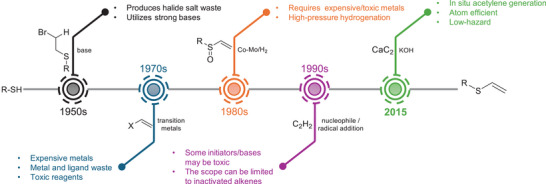
Historical evolution of strategies for constructing vinyl sulfides [20, 21, 22, 23, 24, 25] from thiol precursors. Earlier approaches relied on expensive metals, hazardous reagents, or limited substrate scope, whereas the CaC_2_‐acetylene route enables in situ acetylene generation, atom efficiency, and low‐hazard conditions.

**SCHEME 2:**
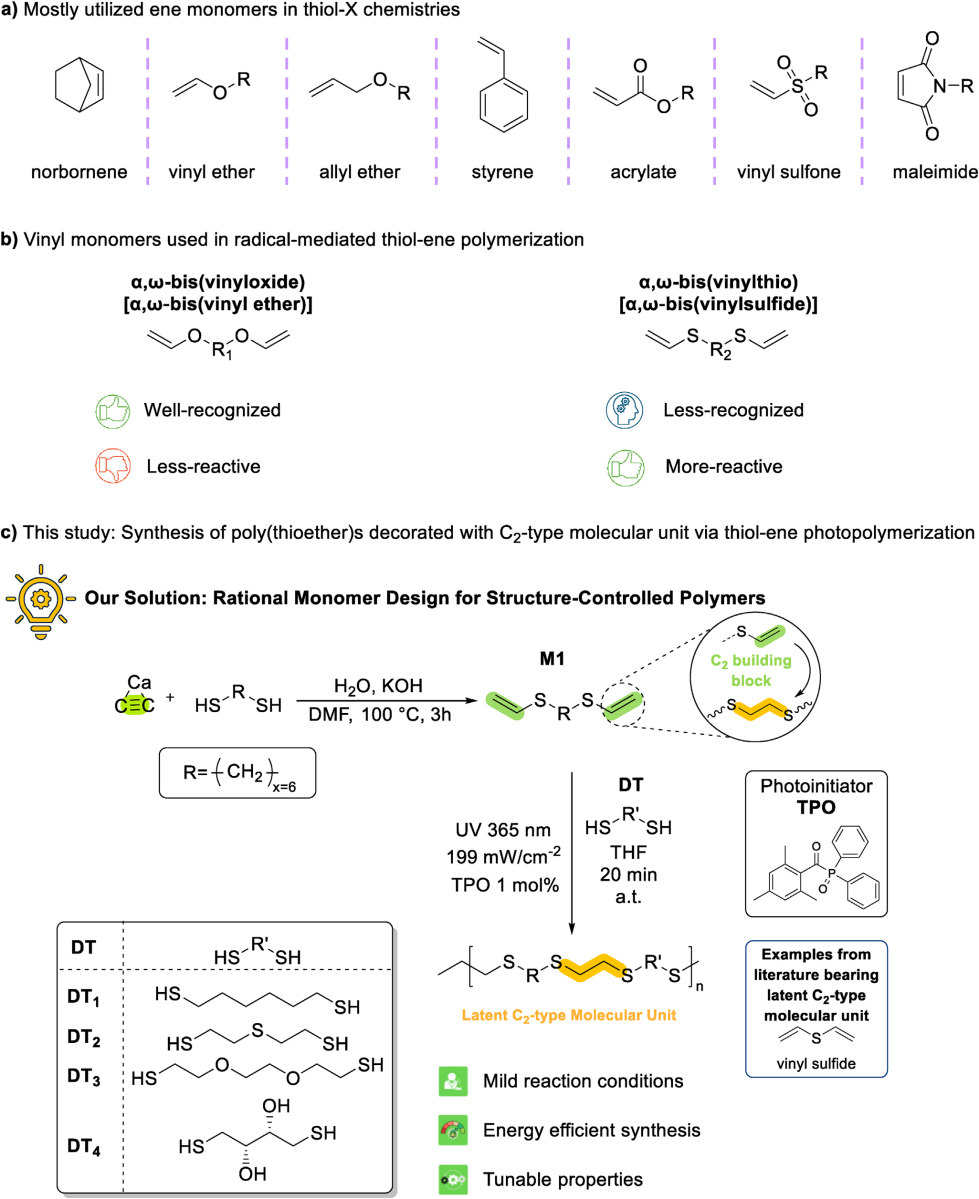
(a) Widely utilized ‘ene’ monomers in thiol‐X step‐growth polymerizations, highlighting norbornene, vinyl ether, allyl ether, styrene, acrylate, vinyl sulfone, and maleimide motifs; (b) Representative vinyl monomers for radical thiol‐ene polymerization, including well‐recognized but less‐reactive α,ω‐bis(vinyloxide), i.e., α,ω‐bis(vinyl ether), and less‐recognized yet more‐reactive α,ω‐bis(vinylsulfide) frameworks; (c) This work: Synthesis of a tailor‐made α,ω‐bis(vinylsulfide) (also known as α,ω‐bis(vinylthio)) monomer (**M1**), and its polymerization with multifunctional thiols to afford poly(thioether)s (**P1**–**P4**) decorated with in situ generated C_2_‐type molecular units via light‐induced radical thiol‐ene step‐growth polymerization.

**FIGURE 1:**
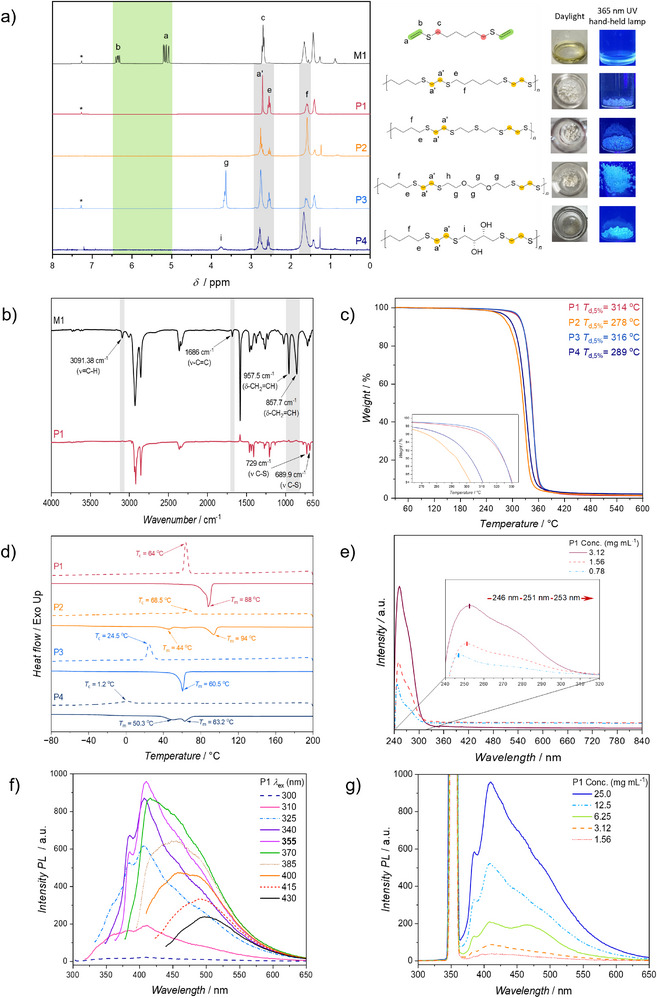
(a) ^1^H NMR spectra (300 MHz, CDCl_3_, 298 K, * denotes residual CHCl_3_) of **M1** (black line), **P1** (purple line), **P2** (green line), **P3** (orange line), **P4** (blue line), and the structures of **P1‐P4** with the C_2_‐spacer highlighted in blue, together with their photographs under daylight and 365 nm hand‐held UV lamp (b) ATR‐IR traces of **M1** (black line) and **P1** (purple line), (c) Comparative TGA thermograms of **P1** (black line), **P2** (yellow line), **P3** (red line), and **P4** (grey line), recorded from 30–600°C at a heating rate of 10 K·min^−1^ under nitrogen flow. (d) Comparative DSC thermograms of polymers **P1** (black line), **P2** (red line), **P3** (blue line), **P4** (green line), and **P5** (grey line). Conditions: The DSC curves are plotted from the first cooling and second heating cycles. Cooling from 200 to −80 °C and second heating from −80 to 200°C, both at 10°C min^−1^ under nitrogen atmosphere, (e) Ultraviolet‐Visible (UV‐Vis) spectra of **P1** at different concentrations (maroon curve: 1.562 mg mL^−1^; orange dash curve: 0.625 mg mL^−1^; blue dash‐dot curve: 0.3125 mg mL^−1^ in CHCl_3_ (298 K). (f) Emission spectra of **P1** recorded at various excitation wavelengths (from 300  to 430 nm, respectively, with an increment (Δλ) of 15 nm) in CHCl_3_ (298 K), at a concentration of 25.0 mg mL^−1^, (g) Emission spectra of **P1** recorded at different concentrations (dark blue curve: 25.0 mg mL^−1^; light blue dash‐dot curve: 12.5 mg mL^−1^; green curve: 6.25 mg mL^−1^; orange dash curve: 3.125 mg mL^−1^; red dot curve: 1.562 mg mL^−1^), at an excitation wavelength of 355 nm in CHCl_3_ (298 K).

**FIGURE 2:**
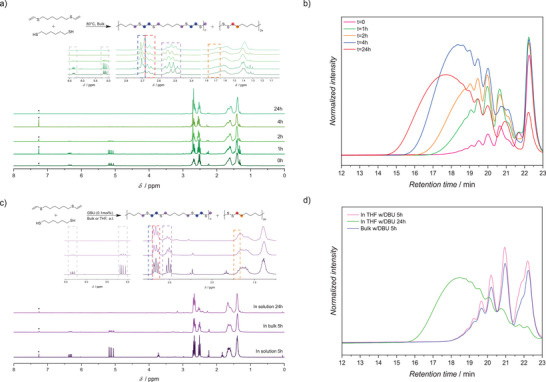
(a) ^1^H NMR spectra (300 MHz, CDCl_3_, 298 K; * denotes residual CHCl_3_) of **M1** during thermally activated catalyst and solvent‐free polymerization at t = 0 h, 1 h, 2 h, 4 h and 24 h  (from bottom to top); (b) Comparative SEC traces of the thermally induced, solvent‐free radical thiol–ene reaction recorded at different time points: t = 0 h (pink), 1 h (green), 2 h (orange), 4 h (blue), and 24 h (red); (c) ^1^H NMR spectra (300 MHz, CDCl_3_, 298 K; * denotes residual CHCl_3_) of the base‐mediated nucleophilic addition utilizing 0.1 mol‐% DBU under different conditions: 5 h in solution, 5 h in bulk, and 24 h in solution (from bottom to top); and (d) Comparative SEC traces of the base‐mediated nucleophilic addition utilizing 0.1 mol% DBU in solution at different time points: t = 5 h in solution (THF, pink), 24 h in solution (green), 5 h solvent‐free (blue). For (a) and (c): The reaction schemes depicted above each spectral set illustrate the corresponding polymerization pathways. The diagnostic regions highlighted by the dashed boxes demonstrate the conversion of vinyl groups into thioether and disulfide functionalities (e.g., purple: α‐protons thioether, blue: β‐protons thioether, red: α‐protons disulfide, and orange: β‐protons disulfide).

**FIGURE 3:**
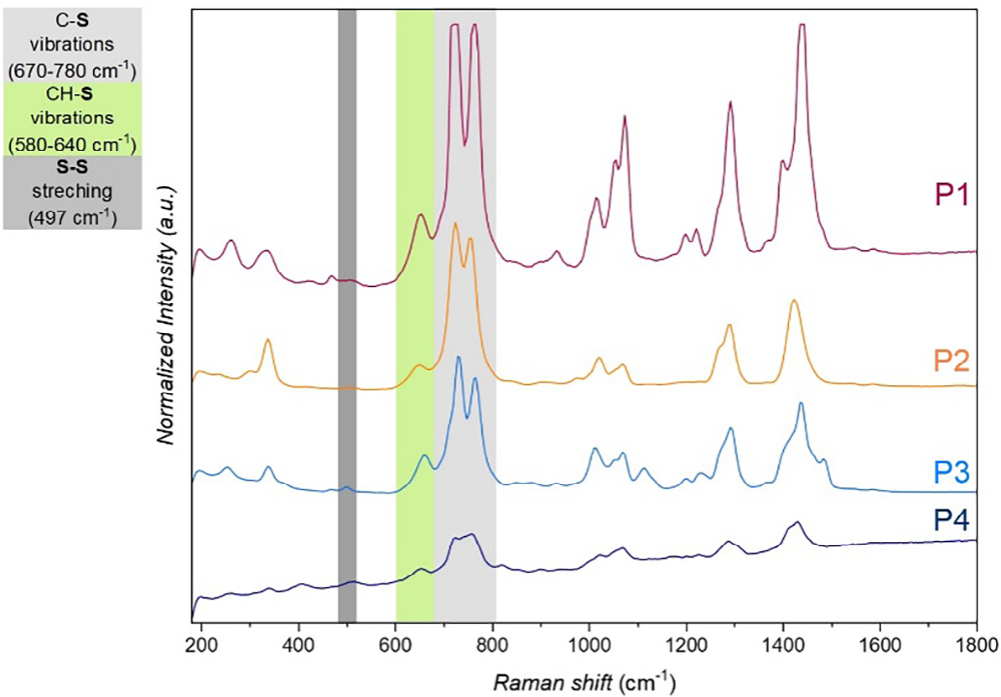
Comparative Raman spectra of **P1**–**P4** recorded at ambient temperature using a dispersive spectrometer (Horiba Labram 300) with 532 nm laser excitation (D03 filter, ∼6 mW at the sample). Acquisition times: **P1**, 30 s; **P2**, 10 s; **P3**, 20 s; **P4**, 3 s.

Thermal characterization was conducted to elucidate how precisely defined C_2_‐spaced thioether linkages and sequence‐defined architecture influence the structural order and stability of the resulting polymers. As aforementioned, thioether‐rich materials are typically associated with soft, amorphous morphologies, owing to flexible C–S–C bonds and weak interchain interactions. However, the introduction of uniform C_2_ spacing and sequence‐defined connectivity was expected to impose regular chain geometry and facilitate close packing. Therefore, thermogravimetric analysis (TGA) and differential scanning calorimetry (DSC) were employed to assess whether this design principle can convert a classically amorphous motif into thermally stable, semi‐crystalline materials. In addition, quantifying decomposition temperatures (*T*_d_,_5%_) would provide insight into the intrinsic robustness of C–S–C frameworks compared to C–C and C–O analogues, while melting and crystallization transitions (*T*_m_ and *T*_c_) would reflect how subtle sequence variations in the dithiol‐derived spacers influence packing and chain cooperativity. The structural diversity of the polymers **(P1‐P4)**, prepared by varying the type of dithiols, was analyzed by TGA and DSC under convenient experimental conditions. As shown by the comparative thermogram and the corresponding transition temperatures (see Figure 1c and Table S1 in the Supporting Information), polymers **P1‐P4** were displaying a single‐step decomposition pattern under nitrogen with *T*_d,5%_ values of 314, 278, 316, and 289°C, respectively. The slightly lower thermal stability of **P2** was attributed to its higher fraction of C–S bonds, which are intrinsically longer and weaker than C–C bonds [43, 44]. Furthermore, **P3** exhibited a decomposition temperature comparable to that of **P1**, despite incorporating ether linkages, suggesting that the sequence‐regular C_2_ backbone compensates through efficient chain organization. The marginally lower decomposition temperature of **P4** likely stems from its hydroxyl‐bearing dithiol precursor, in which polar –OH groups may enhance moisture uptake and promote early degradation. Although TGA does not provide molar mass information, the TGA and Derivative Thermogravimetry (DTG) traces (depicted as Figure S11 in the Supporting Information) exhibited single, well‐defined decomposition steps without early mass loss events, consistent with the absence of substantial low molecular weight oligomers.

The DSC analysis (Figure 1d) has further supported that the C_2_ periodicity and monomer sequence control directly govern the degree of crystallinity. While **P1** has exhibited clear semicrystalline behavior with *T*_m_ ≈ 88°C and *T*_c_ ≈ 64°C, indicative of well‐ordered semicrystalline domains arising from uniform inter‐thioether spacing, **P2** was displaying two melting events (*T*_m_ ≈ 44 and 94°C) with *T*_c_ ≈ 68.5°C, consistent with multiple lamellar populations and the greater sulfur density that stiffens the chain and favors heterogeneous crystallite formation. **P3**, incorporating an oxygen‐containing spacer from 3,6‐dioxa‐1,8‐octanedithiol, has shown a broad and attenuated melting endotherm, indicative of disrupted chain packing due to flexible ether bridges that weaken sequence coherence. In contrast, **P4**, derived from renewable DL‐dithiothreitol, exhibited two broad melting transitions, which have been attributed to microphase‐separated domains arising from hydrophilic –OH groups and hydrophobic segments. This phase heterogeneity has highlighted the sensitivity of the C_2_‐segmented framework to even subtle variations in dithiol polarity and secondary functionality. We note that, beyond the thermal cycling intrinsic to DSC and polarized optical microscopy (POM) image analysis (see discussion below), no dedicated melt‐processing experiments or quantitative mechanical tests were carried out in this study; accordingly, our conclusions are limited to the thermal data and routine laboratory handling of the solid samples.

To explore the crystalline behavior of the obtained materials in greater depth, POM analysis was performed at temperatures ranging from 30 to 90°C (see Figure 4). POM images revealed that **P1** forms a spherulitic morphology on a glass slide upon controlled heating and cooling procedure. The spherulites formed during the heating cycle consists of periodically twisted lamellae (see Figure 4c, d), which persisted up to temperatures close to their melting point (*T*_m_ *=* 88°C). Upon recrystallization (see Figures 4a,b), a transitional morphology is observed. The partial disruption of banding and the increased presence of red elements (see Figure 4e) may indicate the influence of thermal motion on crystallinity. The periodic twisting of ribbon‐like crystals along the radial direction is a hallmark feature of helicoidal spherulites, commonly observed in semi‐crystalline polymers under specific crystallization conditions [45]. Sulfur‐decorated polymers offer non‐conventional luminescent behavior (see Figure 1a, 1e–1g and S19–S23 in the Supporting Information). To explore this phenomenon, the luminescence behavior of **P1** was investigated. Accordingly, the absorption spectra of the solutions (**P1** in CHCl_3_) at different concentrations (from 0.78 mg mL^−1^ to 3.12 mg mL^−1^) were monitored by UV‐Vis spectroscopy (see Figure 1e). As expected, a red shift was noticed (from 246 nm to 252 nm) while the concentration increased (from 0.78 mg mL^−1^ to 3.12 mg mL^−1^), accompanied by two shoulder peaks at 253 and 271 nm. Furthermore, to acquire more information on the emission, a series of fluorescence emission spectra at various concentrations of **P1** was recorded; and it was observed that the photoluminescence (*n*‐PL) intensity consecutively became higher with the increase in concentration (from 1.56 mg mL^−1^ to 25 mg mL^−1^). Complementary to the absorption spectra, two shoulders are evident at 385 and 410 nm (λ_ex_ = 355 nm) (see Figure 1f), indicating the presence of multiple emitting species arising from the thioether moieties in the backbone and conformational rigidification [11]. Additionally, fluorescence emission spectra of **P1** were recorded at various excitation wavelengths (*λ*_ex_ = 300 ‐ 430 nm) at a constant concentration of 25 mg mL^−1^. Correspondingly, emission maximum (*E*_m_) values are noticeably red‐shifted as *λ*_ex_ increases from 300 to 355 nm, as evidenced by the pronounced increase in emission intensity at *λ*_ex_ = 355 nm, reaching approximately 957 a.u. (see Figure 1g). Consequently, the observed behavior of **P1** suggests that it exhibits strong photoluminescence when excited in the UV region (355 nm). For **P1**, the emission intensified with concentration, contrasting the conventional aggregation‐induced quenching (AIQ). Instead, this behavior is consistent with aggregation‐induced emission (AIE), frequently observed in cluster‐triggered emission (CTE) systems [15]. We attribute this behavior to the clustering of thioether functional groups, which enables through‐space conjugation arising from n‐σ* transitions in the C‐S bond, that in turn can interact with nonbonding electron pairs (n) and antibonding σ* orbitals of neighboring bonds without direct overlap. These results have highlighted the tunable optical properties of **P1** and its potential in light‐emitting and sensing applications [11]. Last but not least, the sustainability of the process was evaluated using standard green metrics, including Atom Economy (AE), E‐factor, and the Green Motion tool, which collectively assess synthetic efficiency and environmental impact (see Figure 5) [46], The CaC_2_‐to‐acetylene route, followed by the thiol–yne click to the α,ω‐bis(vinylthio) monomer (**M1**), has demonstrated favorable metrics (AE = 0.71; E‐factor = 8.61, Table S2 and S25 in the Supporting Information), while the subsequent light‐driven thiol–ene polymerization to **P1** (see S24 in the Supporting Information) has achieved near‐perfect Atom Economy (AE = 0.77) and essentially waste‐free conversion (E‐factor ≈ 0). It should be noted that CaC_2_ is produced in an energy‐intensive process and generates carbide‐lime residues, so the overall environmental profile of the route will depend on upstream CaC_2_ sourcing and waste management. Green Motion scoring has further highlighted the high process and reaction efficiency for both steps, with **P1** achieving an overall score of 83 and **M1** of 77. These scores have reflected excellent compliance with several of the Twelve Principles of Green Chemistry, especially in reaction design, process efficiency, and product impact, with room for improvement primarily in raw material sourcing and solvent selection. Overall, these results have emphasized that the presented α,ω‐bis(vinylsulfide) platform not only enables precision polymer synthesis but also supports the development of more sustainable process chemistry.

**FIGURE 4:**
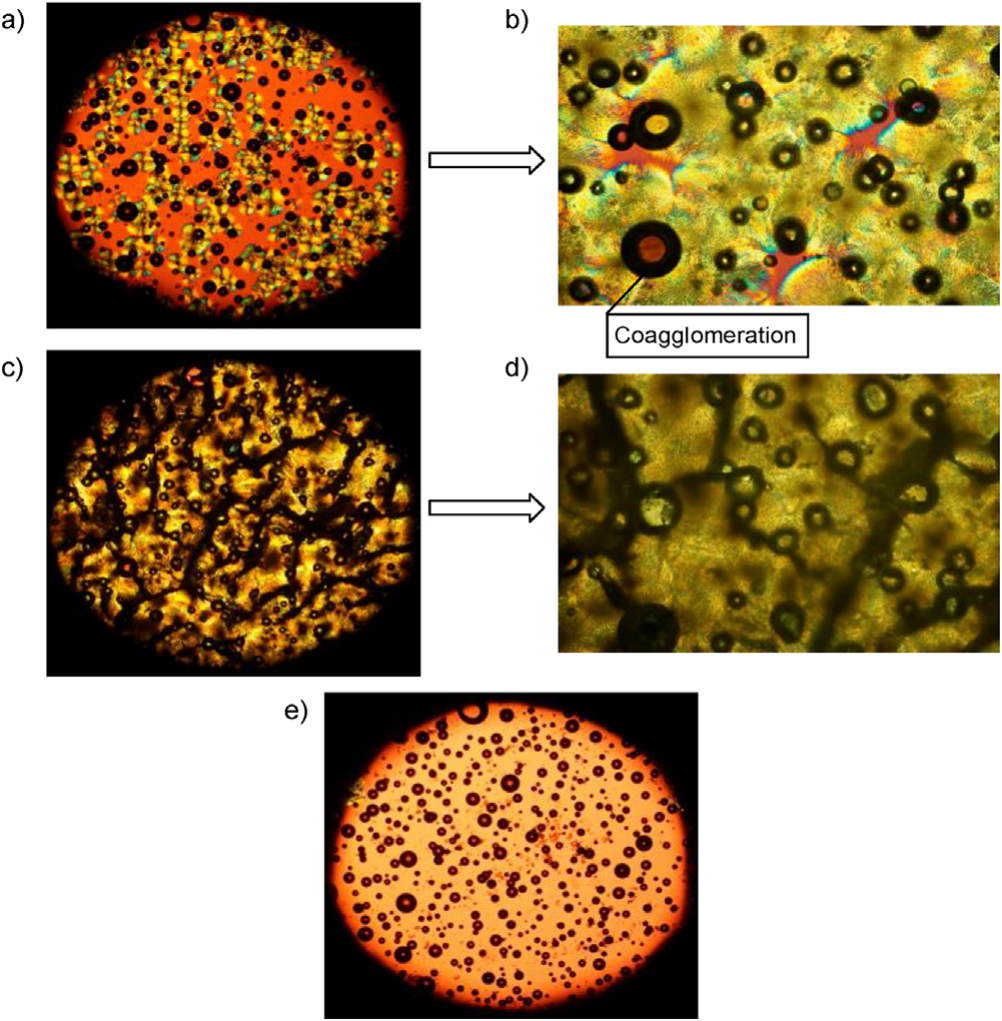
Polarized optical microscopy (POM) images of **P1** spherulites recorded under polarized‐light mode. **P1** was first melted and then imaged during recrystallization (a) Recrystallization at 50°C (2.5×); (b) Recrystallization at 50°C, magnified view (20×); (c) Crystalline State 30°C (2.5×); (d) Crystalline state 30°C, magnified view (20×); (e) Melted state of **P1** prior to recrystallization (2.5×). Scale bars are provided in the Supporting Information, Section B.

**FIGURE 5:**
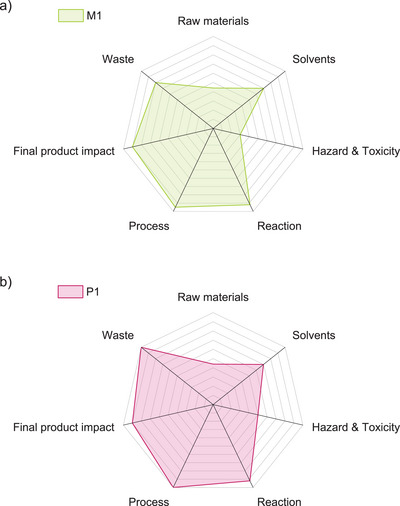
Analysis of the synthesis route of a) **M1** and b) **P1** using the metric tool Green Motion^TM.^

In conclusion, we have established a more‐sustainable and modular platform for the synthesis of sulfur‐decorated polymers, enabled by the preparation of α,ω‐bis(vinylthio), i.e., α,ω‐bis(vinylsulfide), monomer from CaC_2_ via thiol–yne chemistry. This strategy uses CaC_2_‐derived acetylene as a convenient C_2_ building block, thereby bridging molecular design with principles of circular, atom‐efficient and modular chemistry. Systematic exploration of polymerization activation modes has demonstrated that light‐induced thiol–ene polymerization has enabled complete vinyl consumption and the selective formation of anti‐Markovnikov poly(thioether)s. The resulting materials exhibited sharp melting transitions (except **P4**), one‐step thermal decomposition, and cluster‐triggered emission (CTE). These properties are unexpected for thioether‐rich systems, which are traditionally regarded as amorphous and non‐emissive. These unusual characteristics arise from the defined C_2_‐segmented, sequence‐defined backbone, which enforces regular chain spacing and promotes dense thioether clustering capable of through‐space electronic communication. The combination of atom‐efficient monomer synthesis, photochemical selectivity, and sequence control provides a dual advantage: effective use of a readily available C_2_ synthon and access to precision sulfur polymers with tunable thermal and optical properties. Beyond validating the reactivity portfolio of vinyl sulfides, this work has introduced a general design principle for converting soft, flexible linkages into structurally ordered frameworks. Future investigations involving advanced morphological characterization (SAXS, TEM, WAXD) and application‐driven exploration will further expand the technological scope of these C_2_‐engineered poly(thioether)s as more‐sustainable, high‐performance materials [47].

## Conflict of Interests

The authors declare no conflicts of interest.

## Supporting information




**Supporting File 1**: anie71212‐sup‐0001‐SuppMat.pdf.

## Data Availability

The data that support the findings of this study are available in the supplementary material of this article.
