# Supplementary material for: A Dithio Vinylthio C2 Synthon Enabling Crystalline and Luminescent Sulfur‐Decorated Polymers
Source: Angew Chem Int Ed Engl. 2026 Feb 15;65(22):e25801. doi: 10.1002/anie.202525801 (PMC13206596; doi:10.1002/anie.202525801)
Supplement: Supplementary file 1 — Supporting File 1: anie71212‐sup‐0001‐SuppMat.pdf. [file ANIE-65-e25801-s001.pdf]

Supporting Information

A Dithio Vinylthio C_2 Synthon Enabling Crystalline and Luminescent Sulfur-decorated Polymers

*Bercis Pektas[1,2], Cuong Minh Quoc Le[2], Samar Hajjar-Garreau[2], Simon Gree[2], Hatice Mutlu[1,2,3]**

1) Rheinland-Pfälzische Technische Universität Kaiserslautern-Landau, Erwin-Schrödinger-Strasse 52, 67663, Kaiserslautern, Germany
Email: hatice.mutlu@rptu.de
2) Institut de Science des Matériaux de Mulhouse, UMR 7361 CNRS/Université de Haute Alsace, 15 Rue Jean Starcky, Mulhouse Cedex 68057, France
Email: hatice.mutlu@uha.fr
3) Leibniz-Institut für Verbundwerkstoffe (IVW) GmbH
Erwin-Schrödinger-Straße 58, Gebäude 58 / Raum 523, 67663 Kaiserslautern, Germany
Email: hatice.mutlu@leibniz-ivw.de

Content

A. Experimental procedures
1. Materials
2. Monomer synthesis
3. Kinetic Studies
4. Polymer Synthesis
    I. P1
    II. P2
    III. P3
    IV. P4

B. Measurements and analytical methods
1. Nuclear magnetic resonance (NMR) spectroscopy
2. Attenuated total reflectance infrared spectroscopy (ATR-IR)
3. Size exclusion chromatography (SEC)
4. Ultraviolet-Visible Spectroscopy
5. Fluorescence Spectroscopy
6. Thermogravimetric Analysis (TGA)
7. Differential Scanning Calorimetry (DSC)
8. Polarized Optical Microscopy
9. Raman spectroscopy
10. X-ray Photoelectron Spectroscopy (XPS)

C. Additional data and figures

D. Green chemistry metric analysis

E. References

A. Experimental procedures

A.1 Materials

Calcium carbide (Sigma-Aldrich, granulated, technical, ≥75% (gas-volumetric)), 1,6-hexane dithiol (Thermo Scientific Chemicals, 97%), potassium hydroxide (KOH, Prolab, reagent grade), diphenyl(2,4,6 trimethyl benzoyl)phosphine oxide (TPO, Sigma-Aldrich, 97%), 1,8-Diazabicyclo[5.4.0]-7-undecene (DBU, Tokyo Chemical Industry, >98.0%) DL-dithiothreitol (DTT, Sigma-Aldrich, 97%), 2,2′-thiodiethanethiol (TCI, >97%), *n*-Hexane (Thermo Scientific Chemicals, 99%), sodium sulfate (Sigma-Aldrich, ACS reagent, ≥99.0%, anhydrous, powder), *N,N*-Dimethylformamide (DMF, Sigma-Aldrich, ≥99.8%, anhydrous), 3,6-dioxa-1,8-octane-dithiol (EDDT, Sigma-Aldrich, 95%), chloroform stabilized with ethanol (Carlo Erba, 99.99%). Tetrahydrofuran (THF, Sigma-Aldrich, 99%), and methanol (Sigma-Aldrich, ≥99.9%) were anhydrous, and were of HPLC quality and used without further purification. Chloroform D (euriso-top, 99.8%) were used as received.

A.2 Monomer synthesis

Synthetic Procedure for α,ω-bis(vinylsulfide) derivative 1,6-bis(vinylthio)hexane, M1

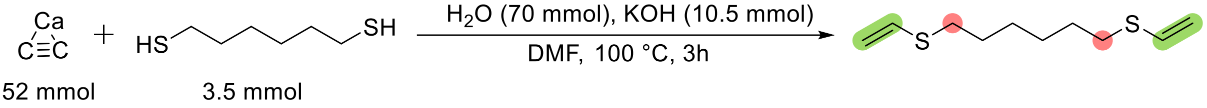

Scheme S1 Synthesis of M1

Potassium hydroxide (0.59 g, 10.5 mmol), 1,6-hexane dithiol (535.170 µL, 3.5 mmol), and dimethylformamide (DMF, 0.175 M, 20 mL) were added to a round-bottom flask. The flask was sealed with a rubber stopper, and the reaction mixture was stirred at an ambient temperature for 20 minutes to ensure complete dissolution of the starting materials. In a separate round-bottomed flask, calcium carbide (4.48 g, 52 mmol) was placed and sealed with a rubber stopper to prevent exposure to ambient moisture. After 20 minutes, the organic reaction flask was heated to 100 °C using a hot block. The two flasks were then connected via a needle to allow the transfer of acetylene gas (as shown in **Scheme S2**).

Distilled water (2.52 mL, 70 mmol) was added incrementally at a controlled rate (0.200 mL per minute) to the flask containing calcium carbide. The gradual addition of water facilitated a steady release of acetylene gas, which was then transferred into the organic reaction mixture. The organic solution, stirred vigorously, changed from colorless to yellow, indicating the progress of the reaction.

After the complete transfer of acetylene gas, the needle connection was removed, and the reaction was maintained at 100 °C for an additional 3 hours. The mixture was then allowed to cool to ambient temperature, followed by a dilution with a 10% aqueous potassium hydroxide solution, and extracted with hexane (3 × 20 mL). The combined organic layers were dried over anhydrous sodium sulfate (Na_2SO_4). The organic solvent was removed under reduced pressure, yielding the crude product as a yellow residue (m = 0.52 g; isolated yield: 73%).

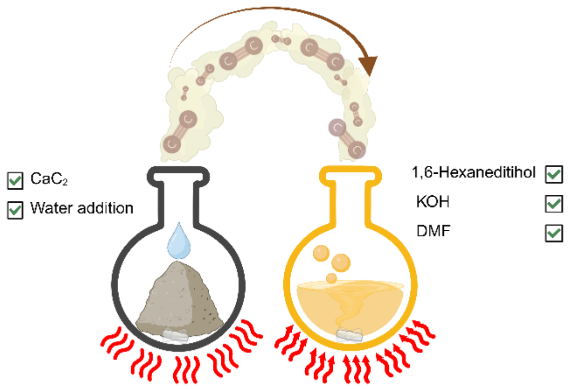

Scheme S2 A typical reaction setup for monomer synthesis.[39]

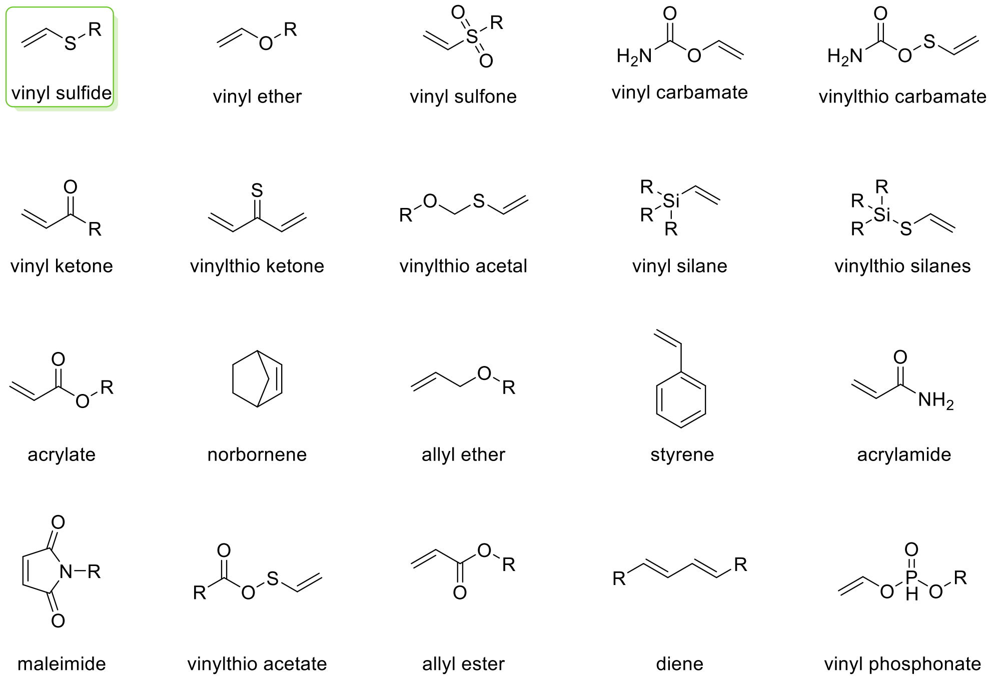

Scheme S3. Comprehensive overview of vinyl-containing C=C motifs explored in thiol–ene reactions to date. The green box highlights this work.

A.3 Kinetic Studies

Thermally-induced solvent-free radical thiol–ene reaction

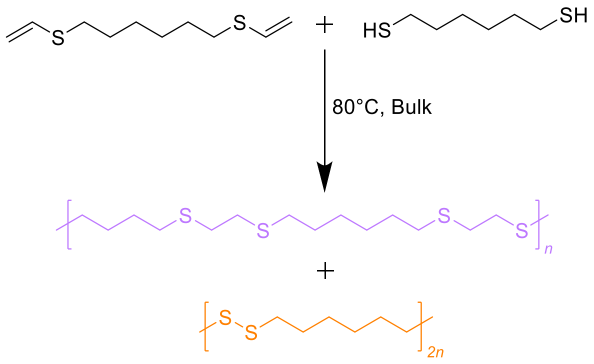

Scheme S4 Synthesis of model polymer via the thermally-induced, solvent-free thiol-ene polymerization at 80°C.

In a 5 mL crimp vial, M1 (0.100 g, 0.494 mmol, 1.00 equiv.) and 1,6-hexanedithiol (0.074 g, 0.494 mmol, 1.00 equiv.) were combined. The vial was placed in a preheated hot block at 80 °C. Aliquots were withdrawn at 1 h, 2 h, 4 h, and 24 h, at which point the reaction was quenched. Each aliquot was analyzed by ^{1}H NMR spectroscopy and size-exclusion chromatography (SEC) to monitor the reaction progress.

Base-mediated thiol–Michael addition

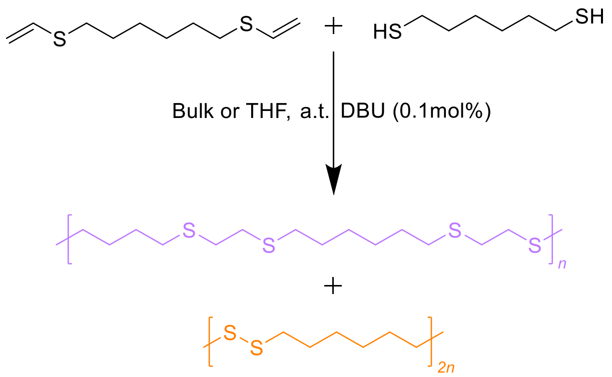

Scheme S5 Synthesis of model polymer via the base-mediated thiol-Michael addition in the presence of 1,8-Diazabicyclo[5.4.0]undec-7-ene (DBU), as the base, at ambient temperature.

a) Solution polymerization: In a 5 mL crimp vial, M1 (0.05 g, 0.247 mmol, ~1.00 equiv.) was dissolved in THF (1 M). Subsequently, 1,6-hexanedithiol (0.036 g, 0.242 mmol, 1.00 equiv.) and DBU (0.1 mol%) were added to the reaction mixture. The reaction was carried out at ambient temperature. Aliquots were withdrawn at 5 h and 24 h. Each aliquot was analyzed by ^{1}H NMR spectroscopy and SEC to monitor the reaction progress.

b) Solvent-free polymerization: In a 5 mL crimp vial, M1 (0.05 g, 0.247 mmol, ~1.00 equiv.), 1,6-hexanedithiol (0.036 g, 0.242 mmol, 1.00 equiv.), and DBU (0.1 mol%) were combined. The reaction was carried out at ambient temperature. After 5 h, the reaction was quenched. The model sample was analyzed by ^{1}H NMR spectroscopy and SEC to monitor the reaction progress.

A.4 Polymer Synthesis

Synthetic procedure of P1

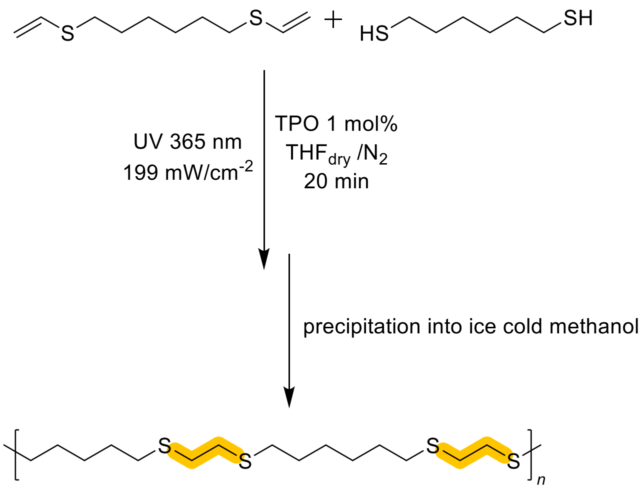

Scheme S6 Synthesis of polymer **P1** via the light-induced thiol-ene photopolymerization in the presence of diphenyl(2,4,6-trimethylbenzoyl)phosphine oxide (TPO) as the photoinitiator at ambient temperature.

In a 5 mL crimp vial, M1 (0.100 g, 0.494 mmol, 1.00 equiv.) was dissolved in dry THF (1 M). Subsequently, 1,6-hexanedithiol (0.074 g, 0.494 mmol, 1.00 equiv.) and TPO (1.0 mol-%) were added to the reaction mixture under a continuous inert atmosphere. The reaction vial was further purged with inert gas, and photopolymerization was conducted at ambient temperature under UV light (365 nm, Hamamatsu, LC-L1V3, 199 mW.cm^{-2}). Upon 20 minutes of light irradiation, the polymer was dissolved in $CHCl_3$ and precipitated into ice-cold methanol to obtain a fine white powder product (m = 0.156 g; isolated yield: 90%).

Synthetic procedure of P2

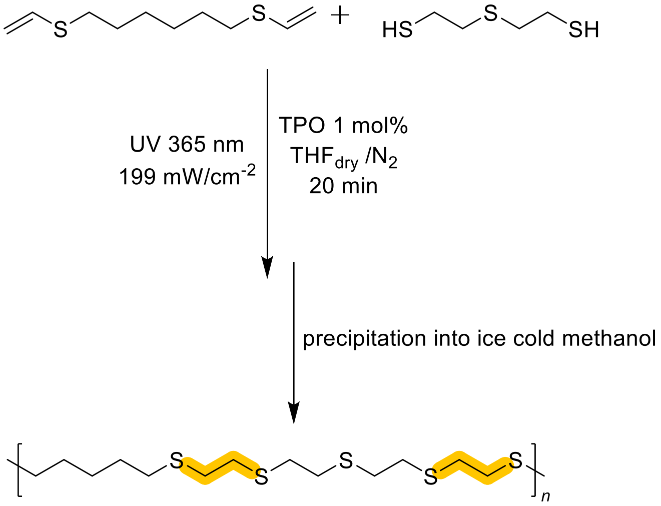

Scheme S7 Synthesis of polymer **P2** via the light-induced thiol-ene photopolymerization in the presence of diphenyl(2,4,6-trimethylbenzoyl)phosphine oxide (TPO) as the photoinitiator at ambient temperature.

In a 5 mL crimp vial, M1 (0.100 g, 0.494 mmol, 1.00 equiv.) was dissolved in dry THF (1 M). Followingly, 2,2′-thiodiethanethiol (0.0762 g, 0.494 mmol, 1.00 equiv.) and TPO (1.0 mol-%) were added into the reaction mixture under a continuous inert atmosphere. The reaction vial was further purged with N_2, and photopolymerization was performed at ambient temperature (365 nm, Hamamatsu, LC-L1V3, 199 mW.cm^{-2}). Upon 20 minutes of light irradiation, the polymer was dissolved in $CHCl_3$ and precipitated into ice-cold methanol to obtain a fine white powder product. (m = 0.1025 g; Yield: 58%)

Synthetic procedure of P3

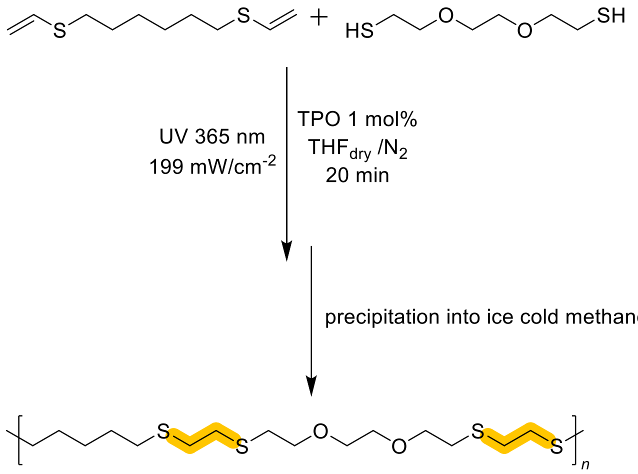

Scheme S8 Synthesis of polymer **P3** via the light-induced thiol-ene photopolymerization in the presence of diphenyl(2,4,6-trimethylbenzoyl)phosphine oxide (TPO) as the photoinitiator at ambient temperature.

In a 5 mL crimp vial, M1 (0.100 g, 0.494 mmol, 1.00 equiv.) was dissolved in dry THF (1 M). Followingly, EDDT (0.090 g, 0.494 mmol, 1.00 equiv.) and TPO (1.0 mol-%) were added into the reaction mixture under a continuous inert atmosphere. The reaction vial was further purged with N_2, and photopolymerization was performed at ambient temperature (365 nm, Hamamatsu, LC-L1V3, 199 mW.cm^{-2}). Upon 20 minutes of light irradiation, the polymer was dissolved in $CHCl_3$ and precipitated into ice-cold methanol to obtain a fine white powder product. (m = 0.08263 g; Yield: 43%)

Synthetic procedure of P4

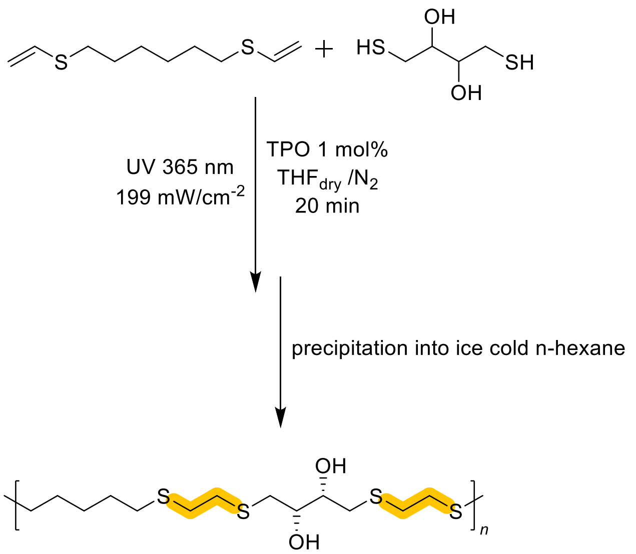

Scheme S9 Synthesis of polymer **P4** the light-induced thiol-ene photopolymerization in the presence of diphenyl(2,4,6-trimethylbenzoyl)phosphine oxide (TPO) as the photoinitiator at ambient temperature.

In a 5 mL crimp vial, M1 (0.100 g, 0.494 mmol, 1.00 equiv.) was dissolved in dry THF (1 M). Followingly, DTT (0.074 g, 0.494 mmol, 1.00 equiv.) and TPO (1.0 mol-%) were added into the reaction mixture under a continuous inert atmosphere. The reaction vial was further purged with N_2, and photopolymerization was performed at ambient temperature (365 nm, Hamamatsu, LC-L1V3, 199 mW.cm^{-2}). Upon 20 minutes of light irradiation, the polymer was dissolved in $CHCl_3$ and precipitated into ice-cold *n*-hexane (x2) to obtain a fine white powder product. (m = 0.152 g; Yield: 86%)

Table S1 Thermal properties of Poly(thioether)s **(P1-P4)** obtained via the light-induced thiol-ene polymerization of M1 in the presence of different dithiol derivatives **(DT_1-DT_4)**.

**Dithiol**[a]	**Polymer**	$T_{d,5\%}$ [°C] TGA[b]	T_m [°C] DSC[c]	T_c [°C] DSC[d]
DT1	P1	314	88	64
DT2	P2	278	44 / 94	68.5
DT3	P3	315.7	60.5	24.5
DT4	P4	289	50.3 / 63.2	1.2

[a] Dithiol derivatives: 1,6-hexanedithiol **(DT_1)**, 2,2'-thiodiethanethiol **(DT_2)**, 3,6-dioxa-1,8-octanedithiol **(DT_3)**, DL-dithiothreitol **(DT_4)**; [b] The decomposition temperature ($T_{d,5\%}$) defined as the temperature at 5% weight loss was detected by TGA; [c] The crystallization temperature (T_c) observed during the first cooling measurement via DSC analysis.[d] The melting temperature (T_c) observed during the second heating measurement via DSC analysis.

B. Measurements and analytical methods

B.1 Nuclear magnetic resonance (NMR) spectroscopy

All ^{1}H-NMR spectra were recorded at least 64 scans on the Varian-300-MR (300 MHz) spectrometer, and ^{13}C-NMR spectra were recorded on the Bruker Avance 500 (125 MHz) spectrometer. The spectra were referenced on the residual solvent signal of $CDCl_3$ according to Nudelman et al. [40]: 7.26 and 77.16 ppm for ^{1}H and ^{13}C, respectively. Deuterated solvents were purchased from Euriso-TOP and used without further purification.

B.3 Attenuated total reflectance infrared spectroscopy (ATR-IR)

All IR measurements were performed on a Bruker Alpha ATR-IR Spectrometer ranging from 650 to 4000 cm^{-1} at ambient temperature.

B.4 Size-exclusion chromatography (SEC)

The apparent weigh average molar mass (M_w) and the molar mass distribution [$Đ$ (dispersity index) = M_w/M_n] values of the polymers were determined using size exclusion chromatography (SEC) measurements on an Agilent 1260 Infinity. The instrument is comprised of an autosampler, a set of columns composed of a guard column (50 × 7.5 mm) and two analytical columns (Polymer Laboratories ResiPore, 300 × 7.5 mm nominal particle size: 3 µm; porosity: 2 µm), G1314B variable wavelength detector operating at 280 nm, a G7800A multidetector suite consisting of a refractive index and a viscosimeter detector. The measurements were conducted with a flow rate of 1 mL min^{-1} at 35 °C using tetrahydrofuran as the eluent. The calibration was carried out by employing different linear poly(styrene) standards (EasiVial polystyrene standards from Agilent) ranging from 162 to 3.64 ×10^5 kDa. The polymer samples were dissolved at a concentration of 2 mg mL^{-1} in the aforementioned eluent and filtered over a 0.2 μm filter prior to the measurement. Agilent GPC/SEC software and multi-detector were used to obtain the apparent molecular weight data.

B.5 Ultraviolet-Visible spectroscopy (UV-Vis)

UV-Vis absorbance was recorded on a V730 UV-vis spectrometer (Jasco Corporation, Japan) operating at 1.0 nm bandwidth and a scan speed of 1000 nm min-1. Monomer or polymer was dissolved in chloroform and transferred to a quartz cuvette (4 × 1 × 1 cm), and spectra were obtained from 235 to 800 nm.

B.6 Fluorescence spectroscopy

Fluorescence spectra were recorded on a FP-8200 (Jasco Corporation, Japan) spectrofluorometer equipped with a xenon lamp. The excitation and emission bandwidths (5 nm) and scan rate (1000 nm min^{-1}) were kept constant. Monomers or polymers were dissolved in a suitable solvent and transferred to a quartz cell (4 × 1 × 1 cm). The spectra were then recorded without degassing.

B.7 Thermogravimetric analysis (TGA)

TGA data were recorded on a Thermogravimetric METTLER - TOLEDO TGA / DSC 3+ from ambient temperature to 600 °C at 30 °C min^{-1} under a nitrogen atmosphere.

B.8 Differential scanning calorimetry (DSC)

A Mettler Toledo DSC1 was used for thermal analysis. The DSC program was set from -80 to 200 °C. Under a nitrogen atmosphere, the heating and cooling rates were 10 °C min^{-1} and 5 °C min^{-1}, respectively.

B.9 **Polarized Light Microscope (POM)**

Crystal growth of the poly(thioether) was observed using a Polarized Optical Microscope (POM, Zeiss Axiophot) with temperature control in the microscope, provided by a Linkam Optical Shearing System (CSS450). The sample for POM observation was used as a crude mixture between clean glass slides, which were heated from 20 °C to 90 °C and then cooled to 30 °C.

B.10 **Raman spectrometry**

The Raman spectroscopy investigation was carried out at a.t. using a dispersive spectrometer Horiba Raman Spectrometer model Labram 300. The excitation was done with a laser at 532 nm (1.74 mW). Rather 1.2 mW

B.10 **X-ray Photoelectron Spectroscopy (XPS)**

X-ray photoelectron spectroscopy (XPS) measurements were done on a VG Scienta SES 2002 spectrometer equipped with a monochromatic Al Kα X-ray source (Al Kα = 1486.6 eV) and an electron gun to compensate the charging effect. The high-resolution spectra and wide scan were recorded with pass energy of 100 eV and 500 eV respectively. The analyzed zone has a surface of 24 mm^2 and an analysis depth of 10 nm. Binding energies (BEs) were calibrated by taking C 1s peak (C-C: 285 eV) of carbon as reference. The peaks were fitted by Gaussian-Lorentzian functions using the CasaXPS software [reference: CasaXPS software, 2.3.26.rev1.0N, Teignmouth, UK] after having subtracted a Shirley-type background.

C. Additional Data and Figures

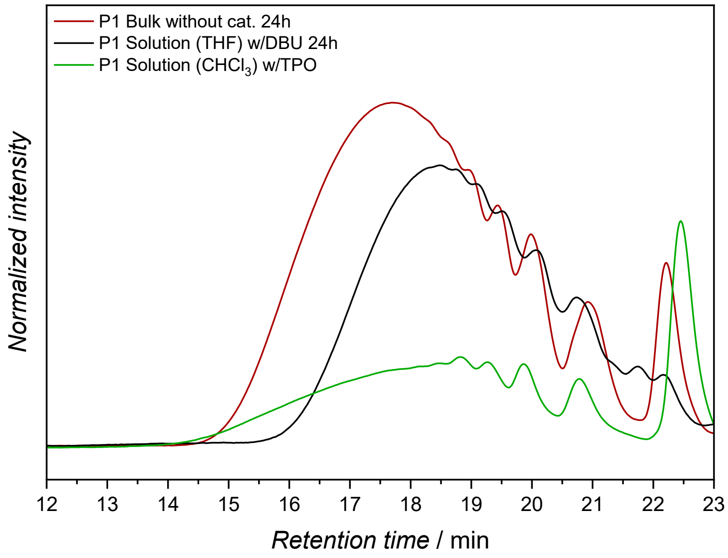

Figure S1. Comparative SEC (with polystyrene standards in THF as an eluent) traces of **P1** obtained under different conditions: thermally induced radical thiol–ene reaction in bulk after 24 h (red), base-mediated thiol–Michael addition in THF in the presence of DBU after 24 h (black), and light-induced radical thiol–ene reaction in $CHCl_3$ in the presence of 1.0 mol-% TPO (green), respectively.

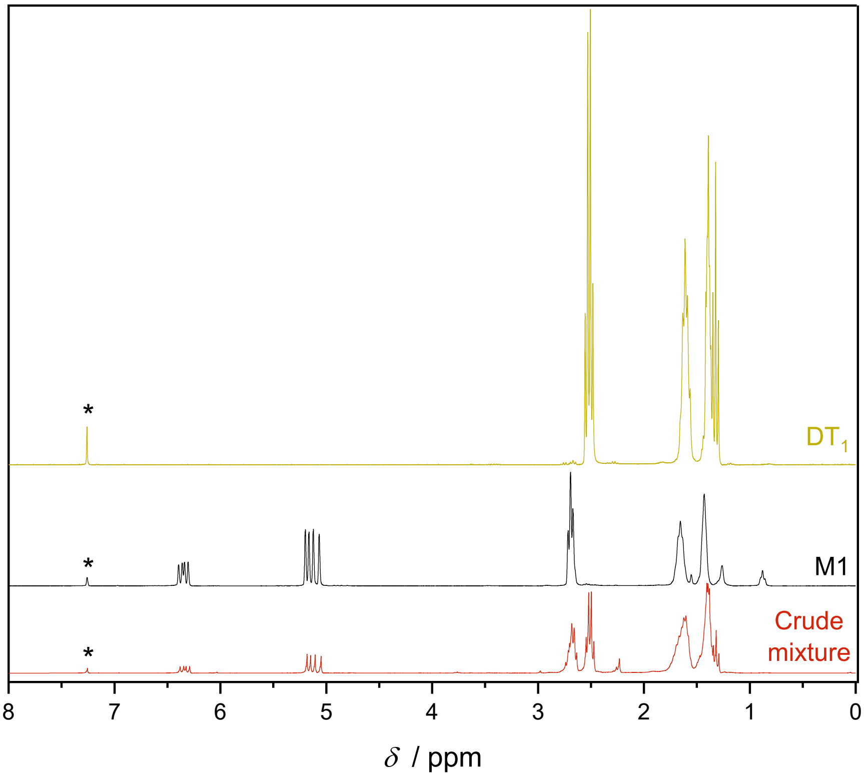

Figure S2. Comparative ^1H NMR spectra of the **DT_1** (khaki), **M1** (black), and the crude reaction mixture of the polymerization (**P1**) (red), respectively.

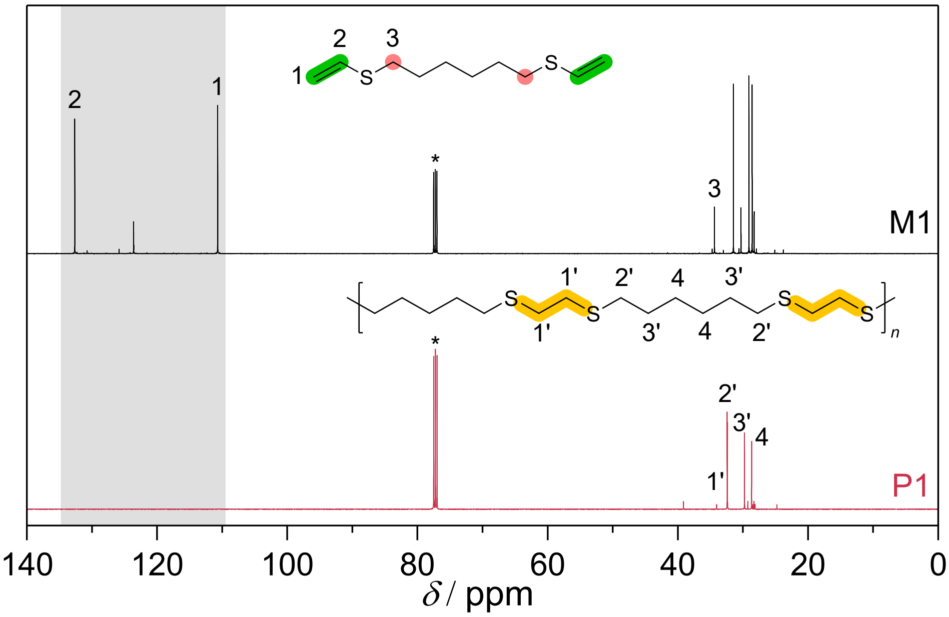

Figure S3 Comparative ^{13}C-NMR (125 MHz, $CDCl_3$) spectra of **M1** (black line) and **P1** (purple line) with residual solvent peaks (*) at ambient temperature. Residual solvents: $CHCl_3$.

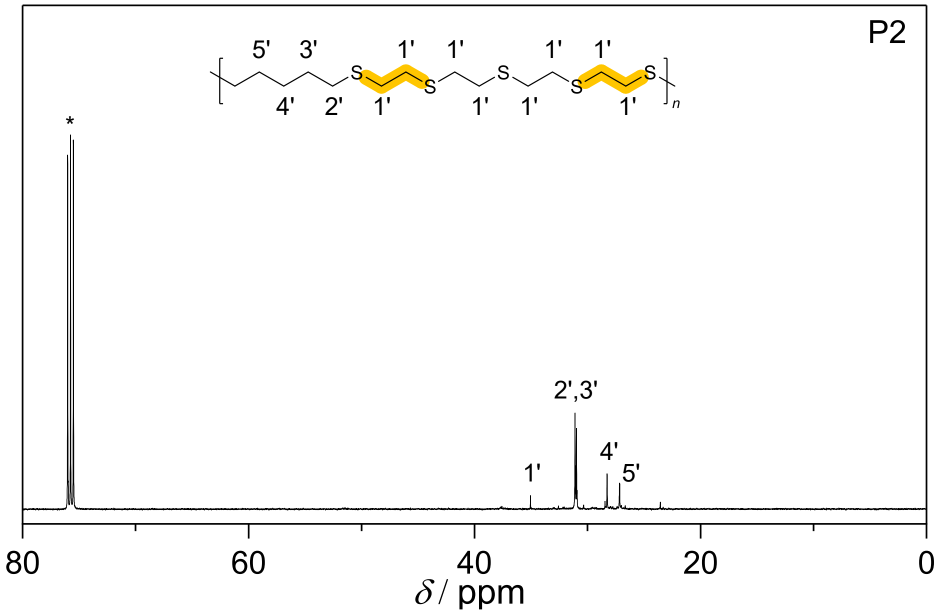

Figure S4 ^{13}C-NMR (125 MHz, $CDCl_3$) spectrum of **P2** with residual solvent peaks (*) at ambient temperature. Residual solvents: $CHCl_3$

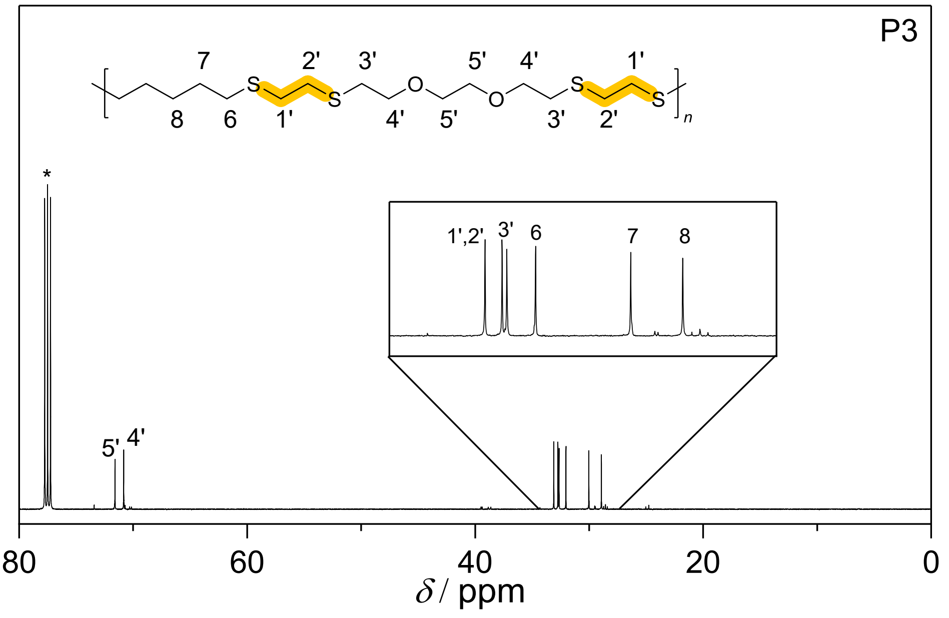

Figure S5 ^{13}C-NMR (125 MHz, $CDCl_3$) spectrum of **P3** with residual solvent peaks (*) at ambient temperature. Residual solvents: $CHCl_3$

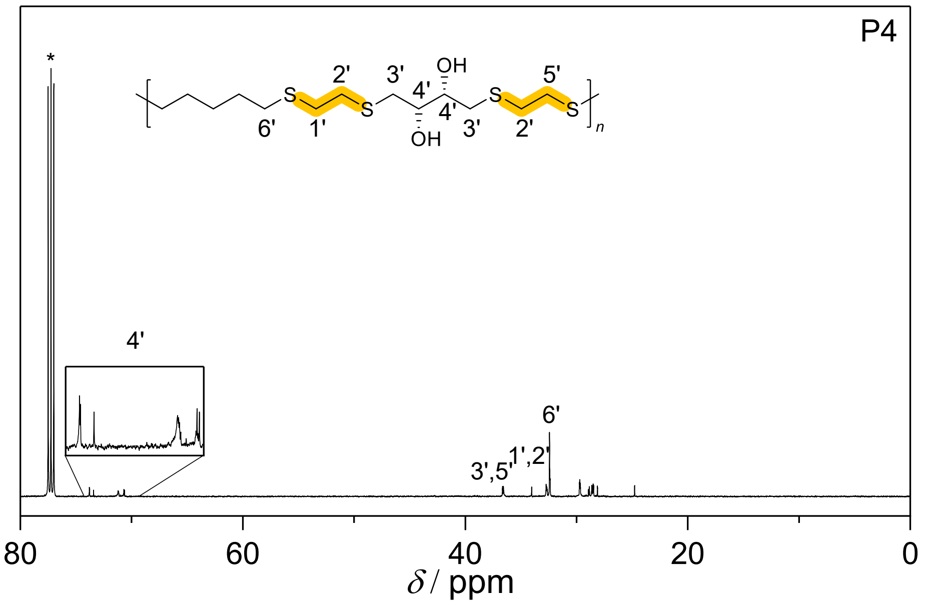

Figure S6 ^{13}C-NMR (125 MHz, $CDCl_3$) spectrum of **P4** with residual solvent peaks (*) at ambient temperature. Residual solvents: $CHCl_3$

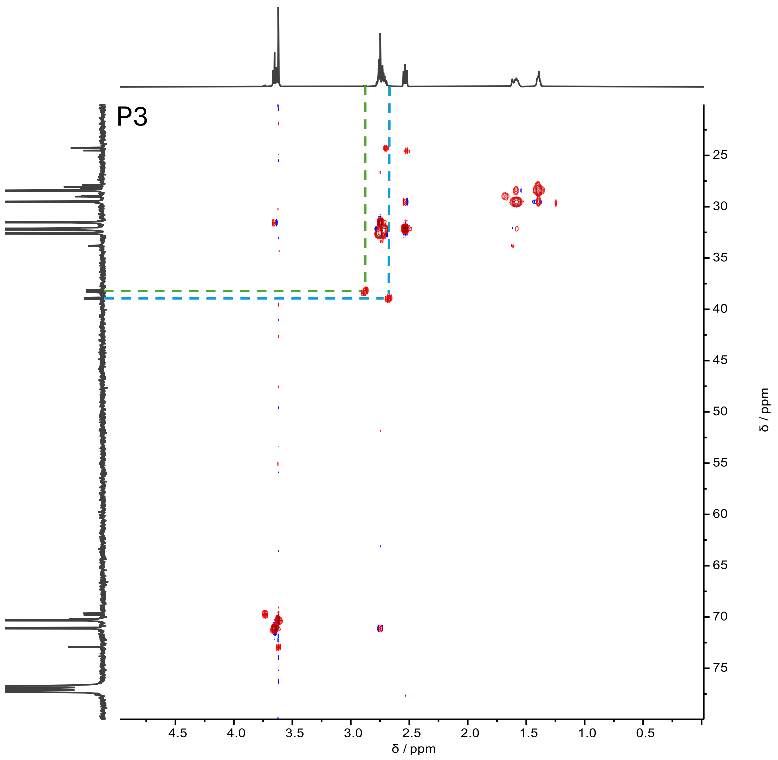

Figure S7 2D HSQC spectrum of **P3** ($CDCl_3$) at ambient temperature

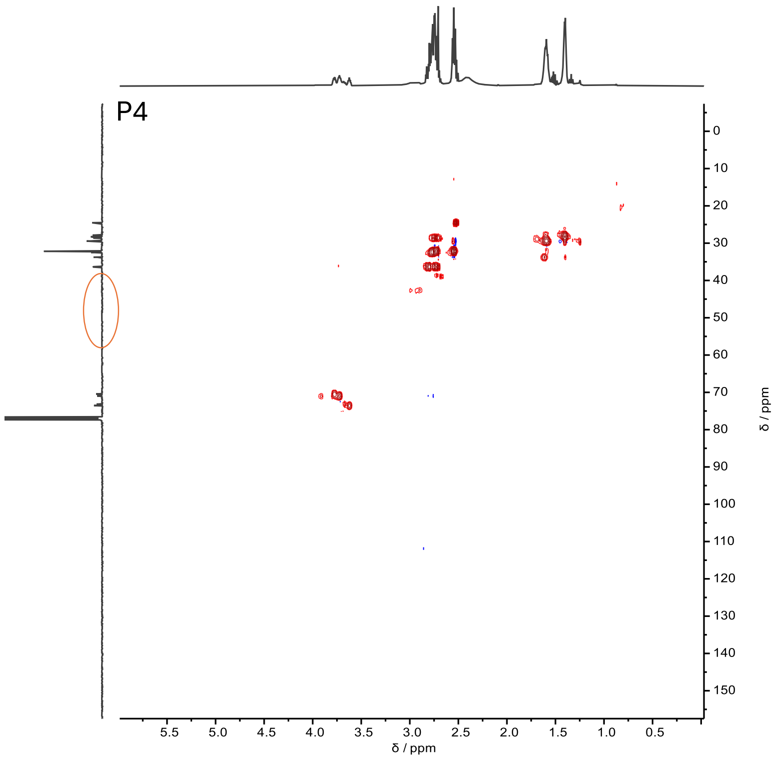

Figure S8 2D HSQC spectrum of **P4** ($CDCl_3$) at ambient temperature highlighting the absence of proton correlations in the region characteristic for C–S–S–C linkages (orange).

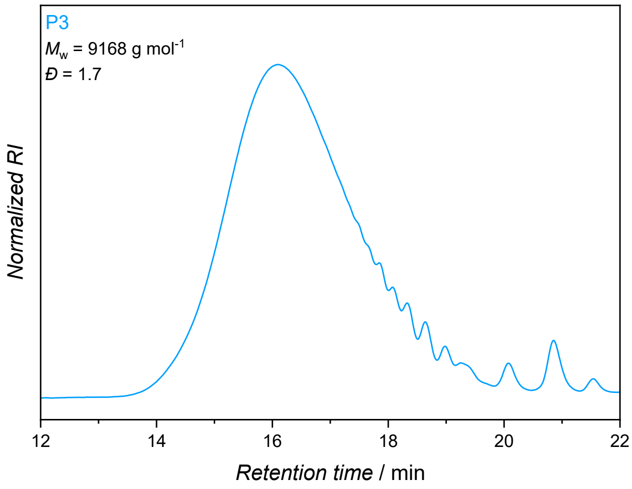

Figure S9 SEC trace of step-growth polymer **P3** (M_w= 9200 g mol^{-1}, Đ = 1.7, blue line) in THF + 0.2% w/v BHT.

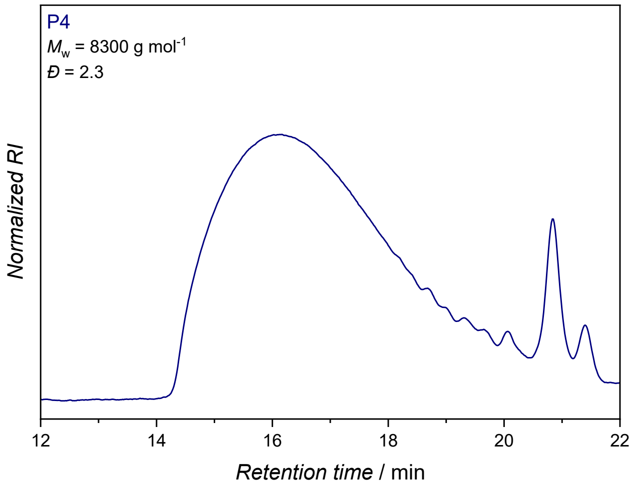

Figure S10 SEC trace of step-growth polymer **P4** (M_w= 8300 g mol^{-1}, Đ = 2.3, navy line) in THF + 0.2% w/v BHT.

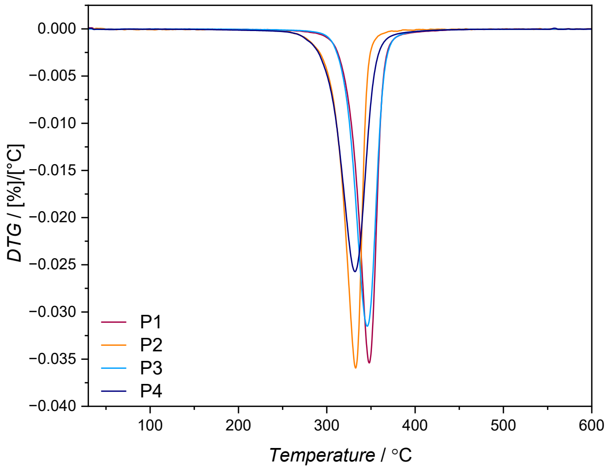

Figure S11 DTG curves of polymers **P1–P4** showing their thermal decomposition behavior as a function of temperature.

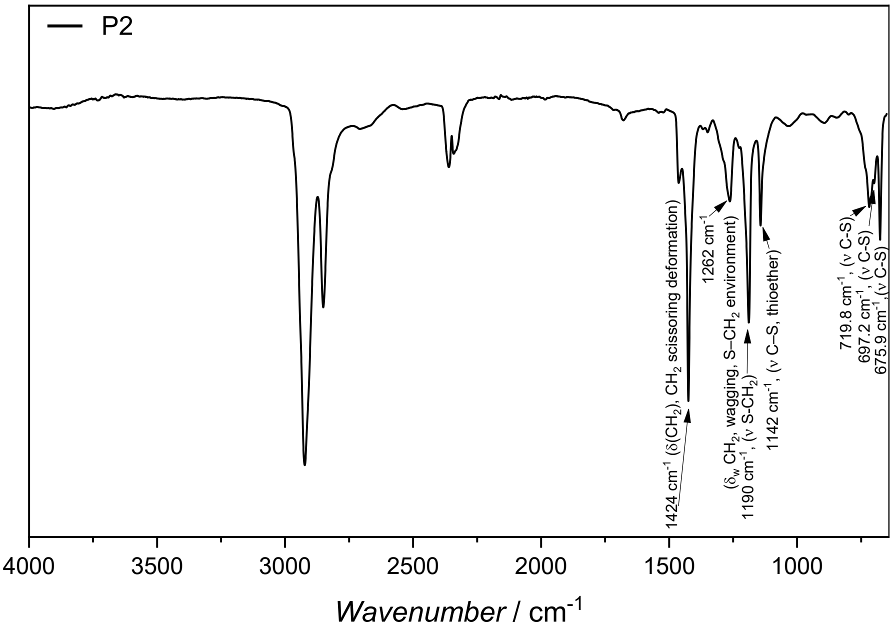

Figure S12 ATR-IR trace of **P2**.

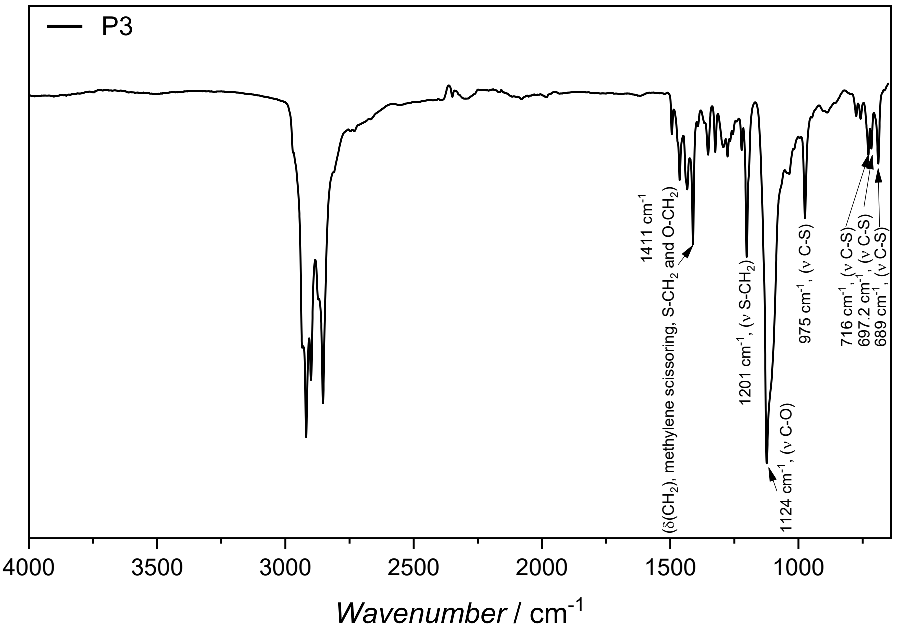

Figure S13 ATR-IR trace of **P3.**

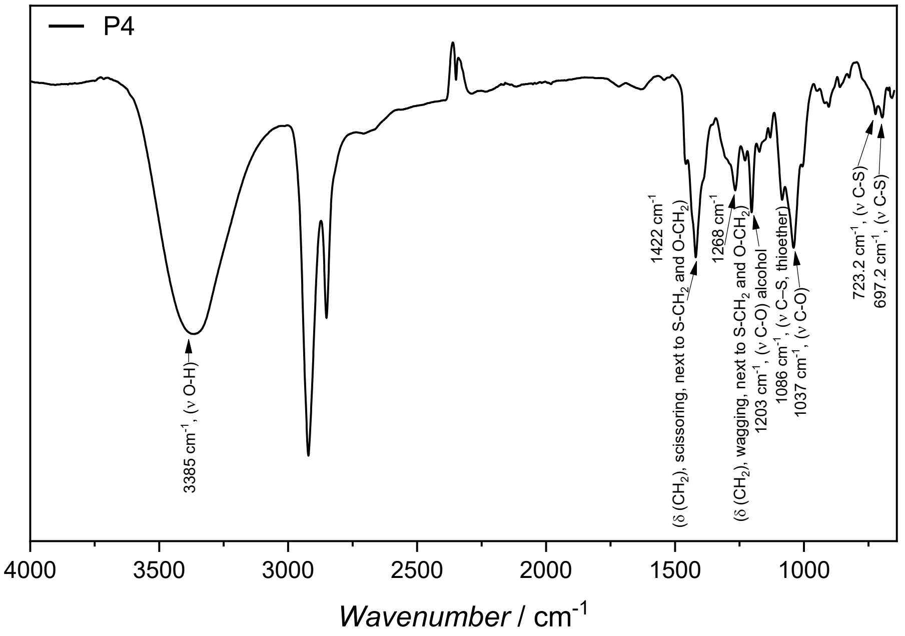

Figure S14 ATR-IR trace of **P4.**

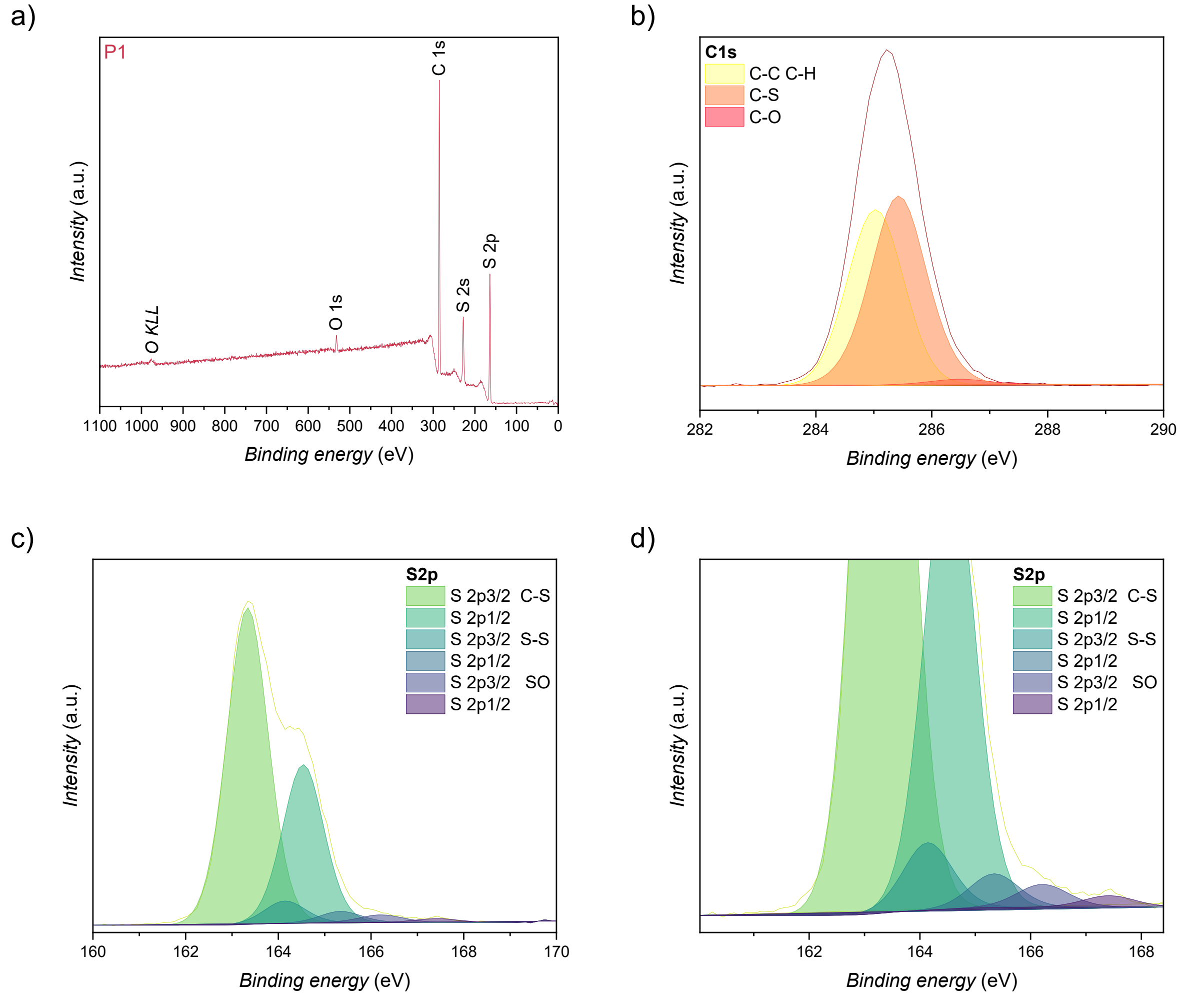

Figure S15 XPS Spectra of **P1 (a)** High resolution XPS spectra of core-levels of **(b)** C 1s, **(c)** S 2p and **(d)** zoom S2p

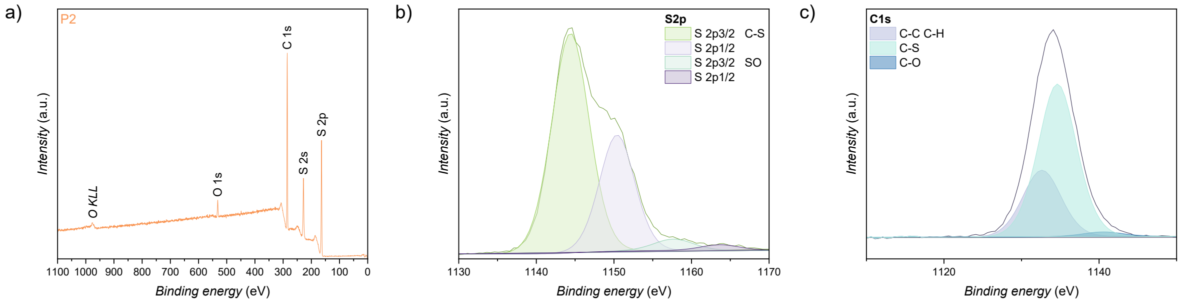

Figure S16 XPS Spectra of **P2** **(a)** High resolution XPS spectra of core-levels of **(b)** C 1s and **(c)** S 2p

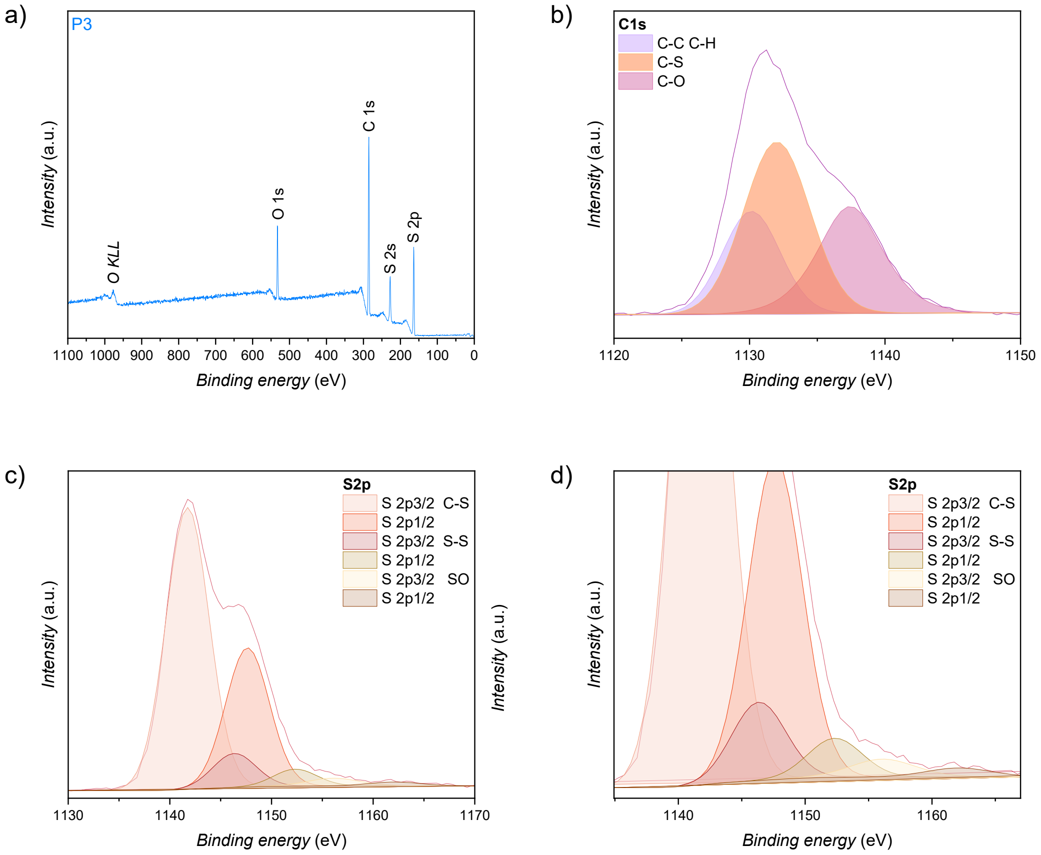

Figure S17 XPS Spectra of **P3** **(a)** High resolution XPS spectra of core-levels of **(b)** C 1s, **(c)** S 2p, and **(d)** zoom S2p

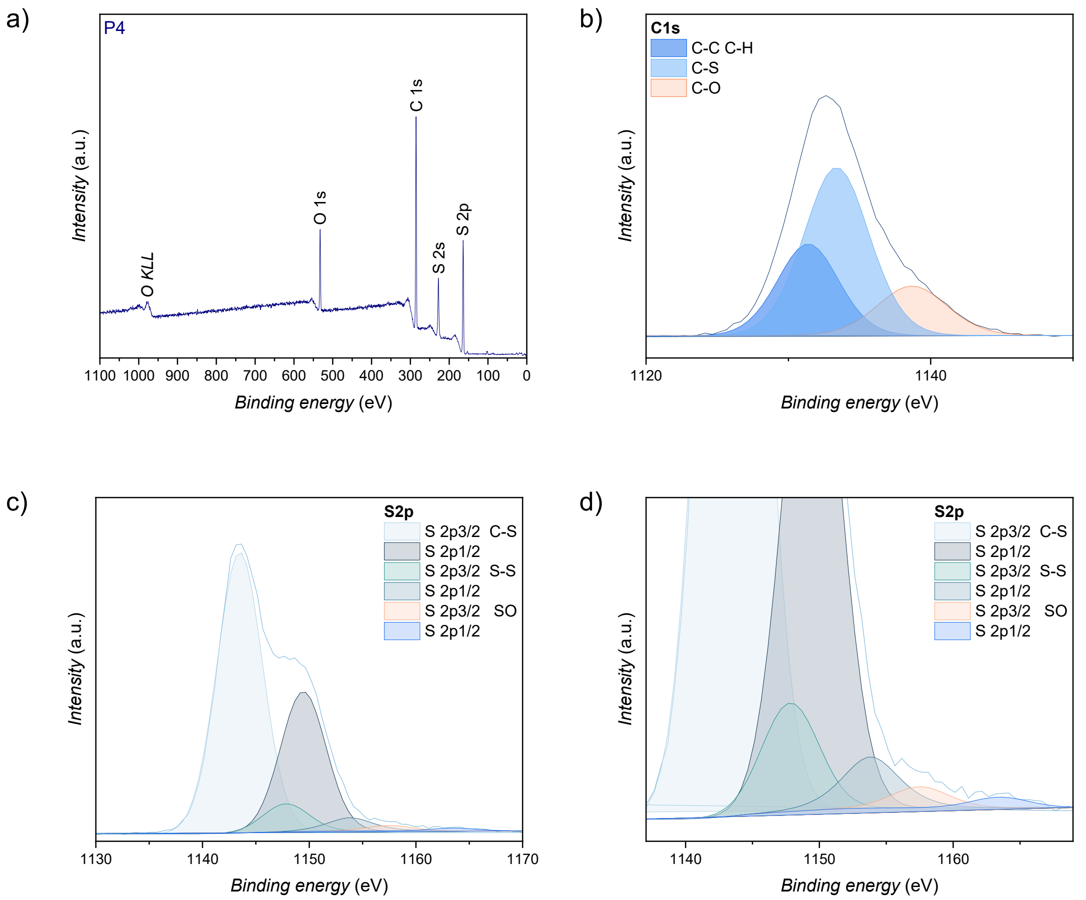

Figure S18 XPS Spectra of **P4** **(a)** High resolution XPS spectra of core-levels of **(b)** C 1s, **(c)** S 2p, and **(d)** zoom S2p

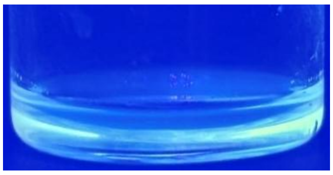

Figure S19 M1 under irradiation with a UV hand-held lamp at 365 nm.

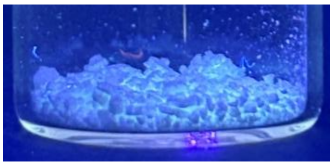

Figure S20 P1 under irradiation with a UV hand-held lamp at 365 nm.

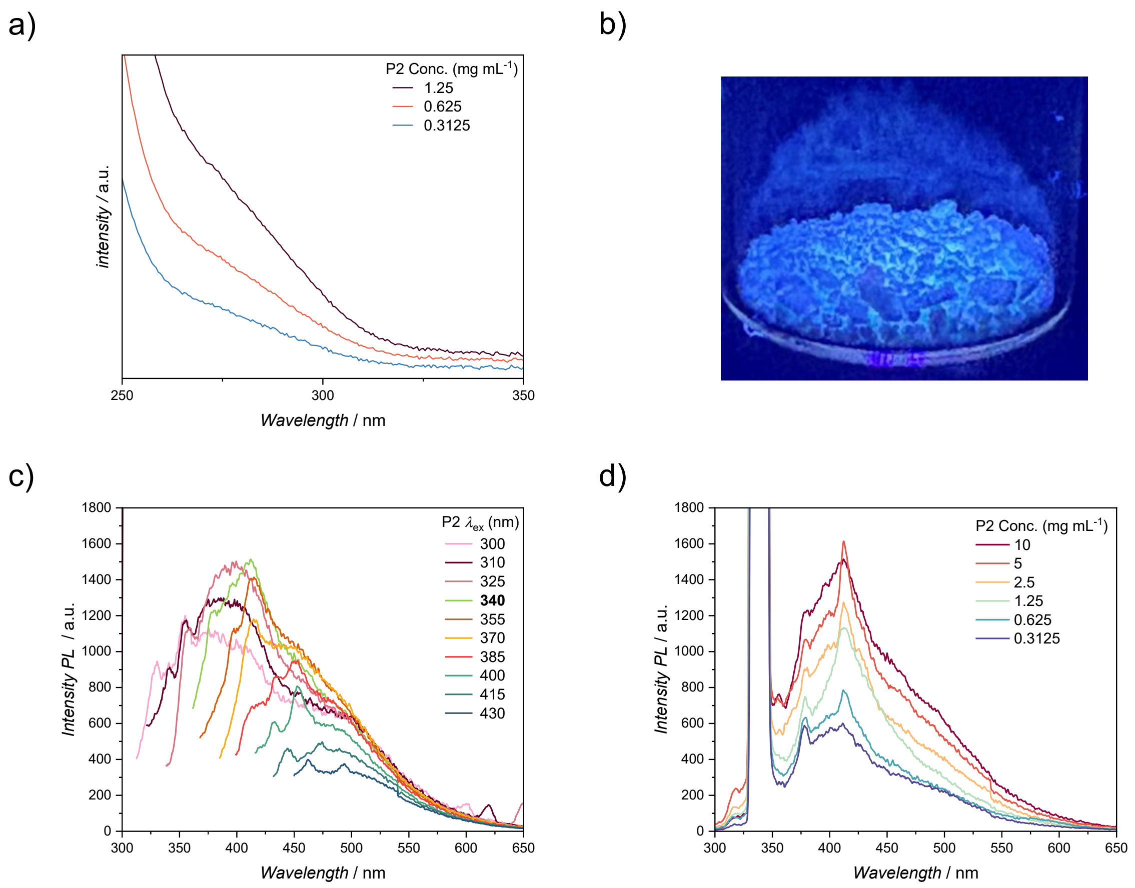

Figure S21 (a) Ultraviolet-visible (UV-Vis) spectra of **P2** at different concentrations (maroon curve: 1.25 mg mL^{-1}; red curve: 0.625 mg mL^{-1}; blue curve: 0.3125 mg mL^{-1} in $CHCl_3$ (298 K); **(b) P2** under irradiation under a UV hand-held lamp at 365 nm; **(c)** Emission spectra of **P2** at various excitation wavelengths (from 300 nm to 430 nm, respectively, with an increment of 15 nm in $CHCl_3$ (298 K), at a concentration of 10 mg mL^{-1}; **(d)** Emission spectra of **P2** at different concentrations (burgundy curve: 10 mg mL^{-1}; red curve: 5 mg mL^{-1}; orange curve: 2.5 mg mL^{-1}; green curve:1.25 mg mL^{-1}; blue curve: 0.625 mg mL^{-1}; dark blue curve:0.3125 mg mL^{-1}), at an excitation wavelength of 340 nm in $CHCl_3$ (298 K).

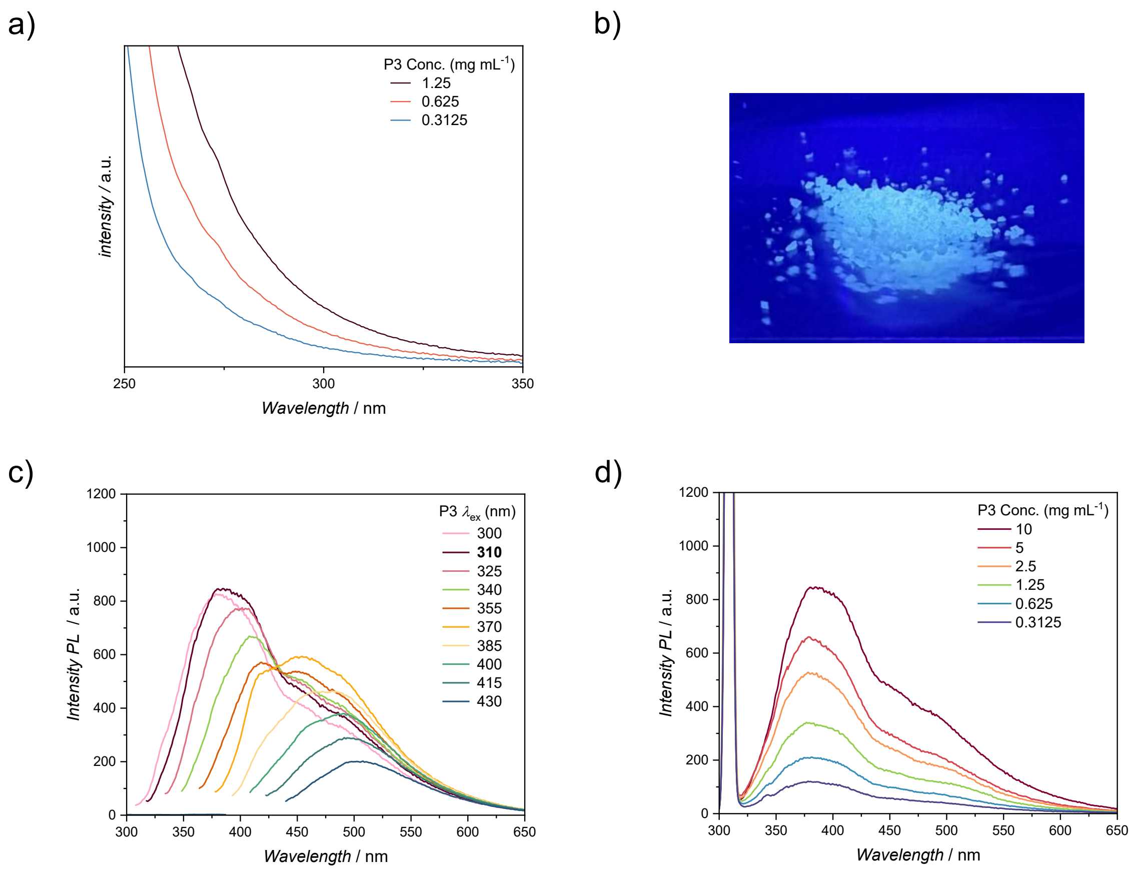

Figure S22 (a) Ultraviolet-visible (UV-Vis) spectra of **P3** at different concentrations (maroon curve: 1.25 mg mL-1; red curve: 0.625 mg mL-1; blue curve: 0.3125 mg mL^{-1} in CHCl3 (298 K); **(b)** P3 under irradiation with a UV hand-held lamp at 365 nm; **(c)** Emission spectra of **P3** at various excitation wavelengths (from 300 nm to 430 nm, respectively, with an increment of 15 nm in CHCl$_3$ (298 K), at a concentration of 10 mg mL^{-1}; **(d)** Emission spectra of **P3** at different concentrations (burgundy curve: 10 mg mL^{-1}; red curve: 5 mg mL^{-1}; orange curve: 2.5 mg mL^{-1}; green curve:1.25 mg mL^{-1}; blue curve: 0.625 mg mL^{-1}; dark blue curve:0.3125 mg mL^{-1};), at an excitation wavelength of 310 nm in CHCl$_3$ (298 K).

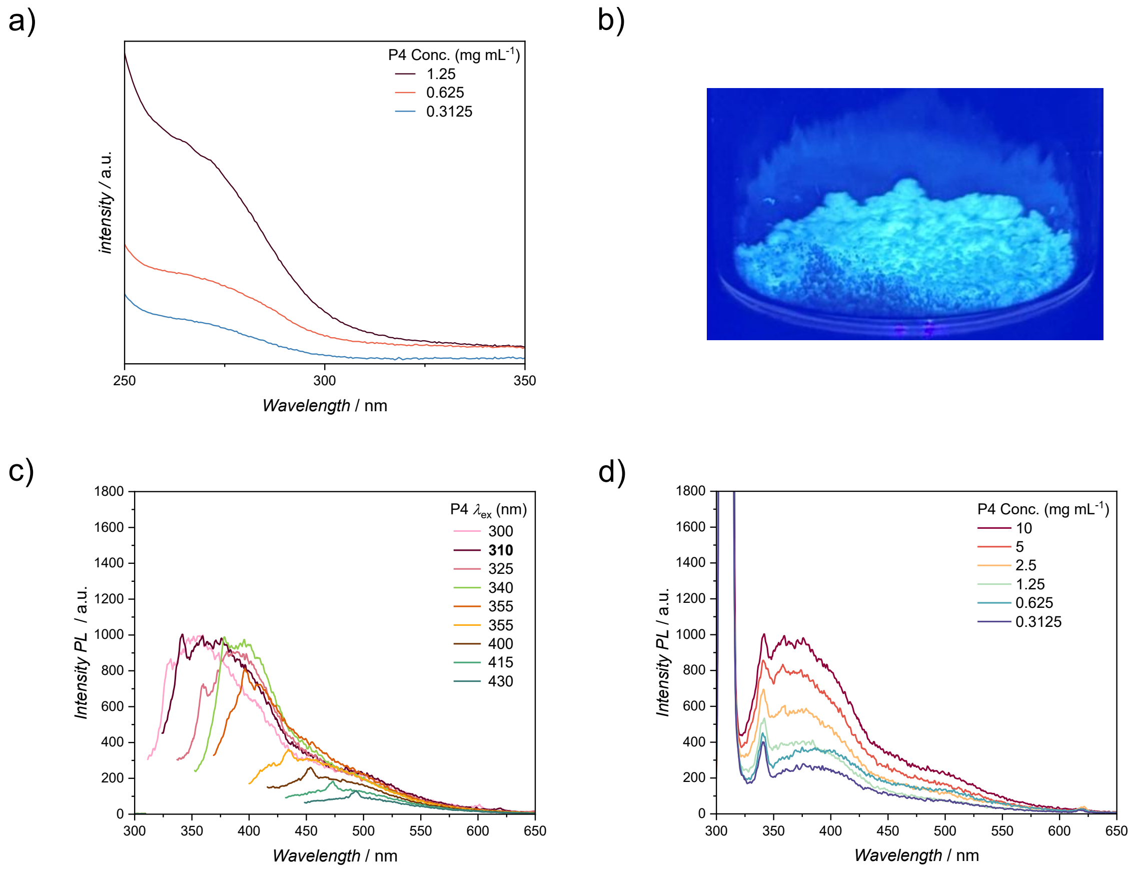

Figure S23 **(a)** Ultraviolet-visible (UV-Vis) spectra of **P4** at different concentrations (maroon curve: 1.25 mg mL^{-1}; red curve: 0.625 mg mL^{-1}; blue curve: 0.3125 mg mL^{-1} in $CHCl_3$ (298 K); **(b) P4** under irradiation with a UV hand-held lamp at 365 nm; **(c)** Emission spectra of **P4** at various excitation wavelengths (from 300 nm to 430 nm, respectively, with an increment of 15 nm in $CHCl_3$ (298 K), at a concentration of 10 mg mL^{-1}; **(d)** Emission spectra of **P4** at different concentrations (burgundy curve: 10 mg mL^{-1}; red curve: 5 mg mL^{-1}; orange curve: 2.5 mg mL^{-1}; green curve:1.25 mg mL^{-1}; blue curve: 0.625 mg mL^{-1}; dark blue curve:0.3125 mg mL^{-1};), at an excitation wavelength of 310 nm in $CHCl_3$ (298 K).

D. Green chemistry metrics analysis [41,42]

The following formulas were used to calculate the E-factor and Atom Economy (AE).

$$\text{E-factor} = \frac{Total\ mass\ of\ waste}{Mass\ of\ product}$$

$$\text{Atom Economy} = \frac{\text{Number of carbons of the product at the end of the step}}{Total\ number\ of\ carbons\ in\ the\ main\ reagents\ involved}$$

Table S2. Quantitative assessment of environmental impact and synthetic efficiency for **M1** and **P1**

Quantitative data / Product name	E-factor	Atom Economy
**M1**	8.610	0.710
**P1**	0	0.77

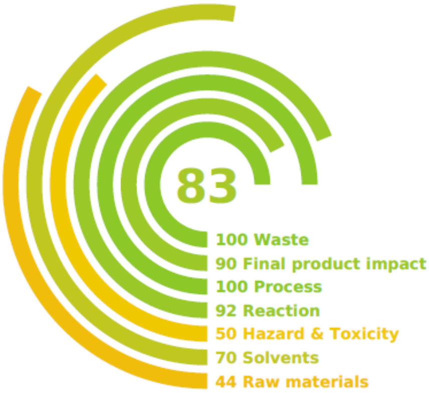

Figure S24 Representative scheme summarizing the assessment of the environmental impact of **P1** manufacture according to the 12 principles of Green Chemistry.

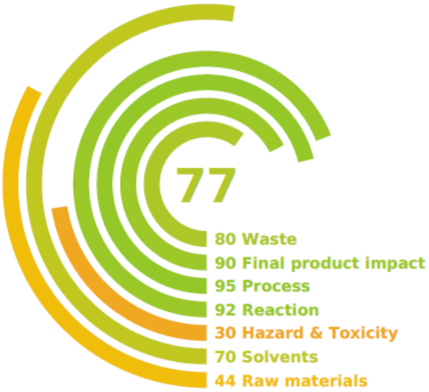

Figure S25 Representative scheme summarizing the assessment of the environmental impact of **M1** manufacture according to the 12 principles of Green Chemistry.

References

[39] Created in BioRender. Pektas, B. (2025) https://BioRender.com/r58i009
[40] G. R. Fulmer, A. J. M. Miller, N. H. Sherden, H. E. Gottlieb, A. Nudelman, B. M. Stoltz, J. E. Bercaw, K. I. Goldberg, *Organometallics*, **2010**, *29*, 2176.
[41] Phan, T. T., Gallardo, C., & Mane, J, *Green Chem.*, **2015**, 17(5), 2846-2852.
[42] https://greenmotion.mane.com/
